# Supplementary material for: Chad Genetic Diversity Reveals an African History Marked by Multiple Holocene Eurasian Migrations
Source: Am J Hum Genet. 2016 Nov 23;99(6):1316–24. doi: 10.1016/j.ajhg.2016.10.012 (PMC5142112; doi:10.1016/j.ajhg.2016.10.012)
Supplement: Document S1. Figures S1–S6 and Tables S1–S6 [file mmc1.pdf]

The American Journal of Human Genetics, Volume *99*

Supplemental Data

Chad Genetic Diversity Reveals an African History Marked by Multiple Holocene Eurasian Migrations

Marc Haber, Massimo Mezzavilla, Anders Bergström, Javier Prado-Martinez, Pille Hallast, Riyadh Saif-Ali, Molham Al-Habori, George Dedoussis, Eleftheria Zeggini, Jason Blue-Smith, R. Spencer Wells, Yali Xue, Pierre A. Zalloua, and Chris Tyler-Smith

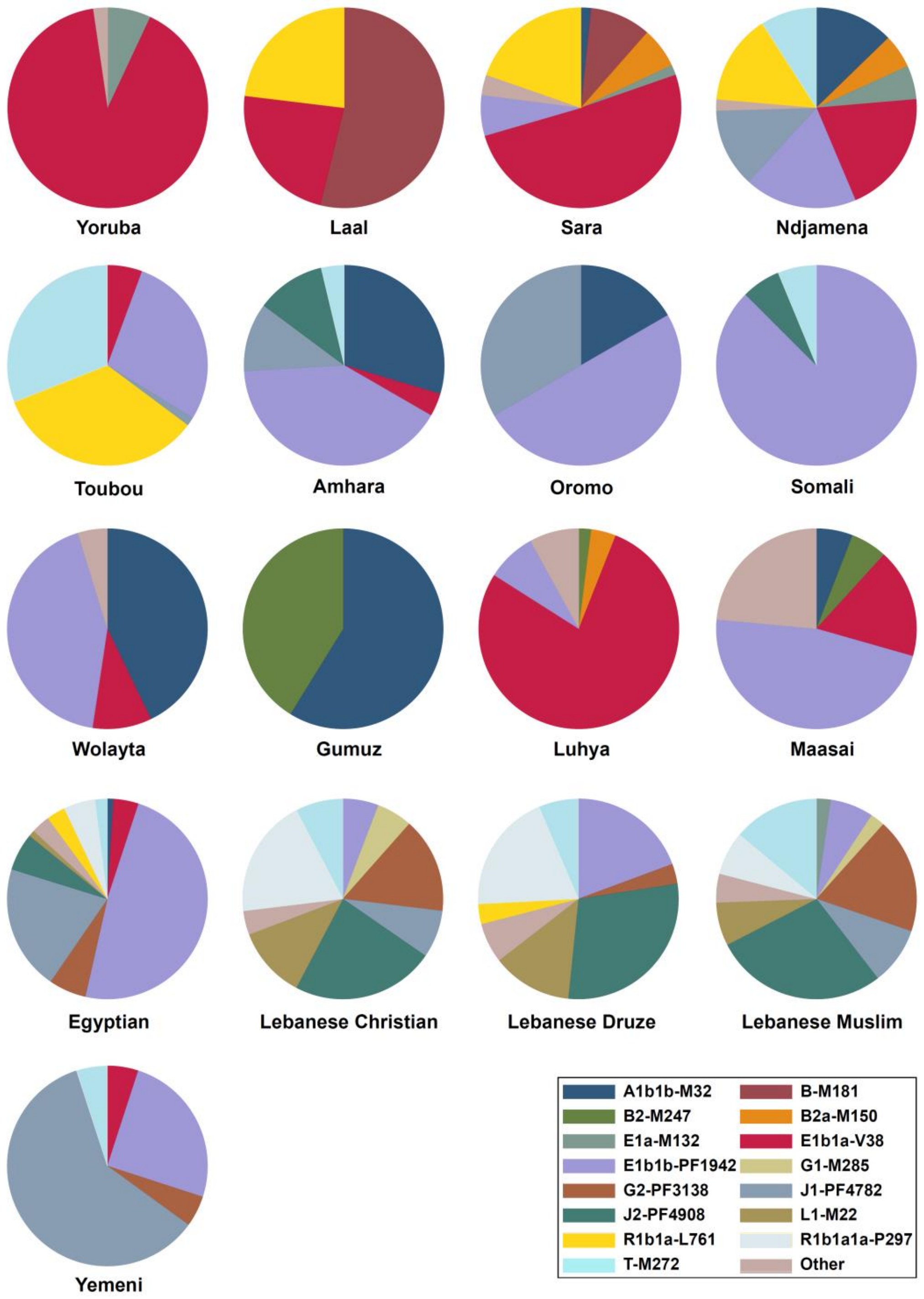

Figure S1. Y-chromosome haplogroup distribution. We find in Chad a high frequency of Y-haplogroup R1b in all ethnic groups examined. On the other hand, R1b is absent or rare in East African populations. We also find the Eurasian haplogroup T in Toubou and Ethiopians with Toubou having a high frequency (31%) of their studied males belonging to this haplogroup. Interestingly, the only instances of this haplogroup in examined ancient populations are in the *Linearbandkeramik* (LBK) population which we found to be the most significant reference for the Eurasian ancestry in Toubou and the Ethiopians. For more detailed haplogroup definitions see Table S2. Category "Other" contains haplogroups B2b, E, E2, I2, Q, R1a and R2a.

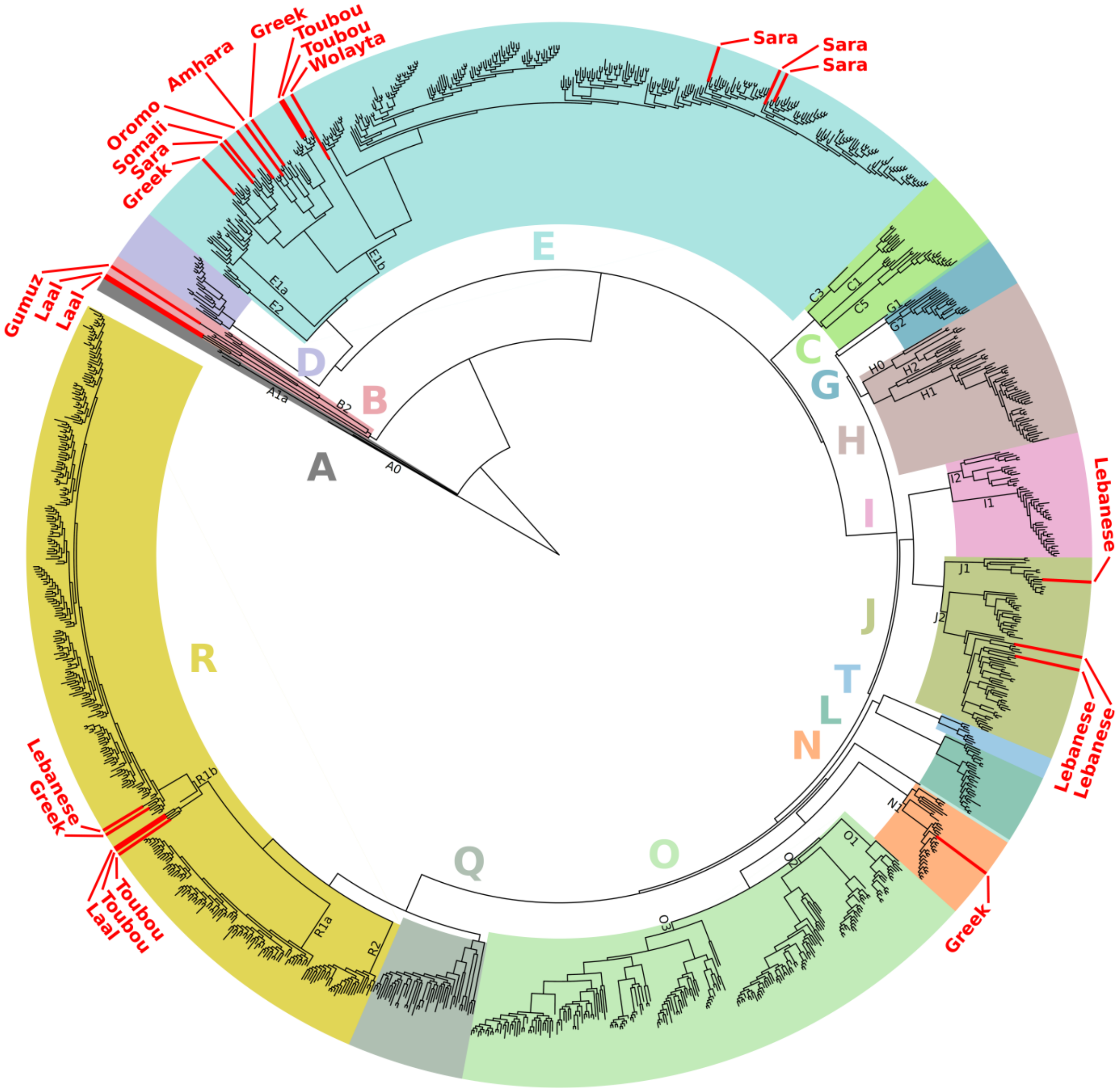

Figure S2. Y-chromosome sequences in a global phylogenetic context. A maximum likelihood phylogenetic tree of the sequenced Y chromosomes in our dataset and those of all the 1244 males from the 1000 Genomes Project. Coloured ranges correspond to high level haplogroups. Some finer-scale haplogroup information is indicated by labels on the tree. The samples sequenced in this study are highlighted in red.

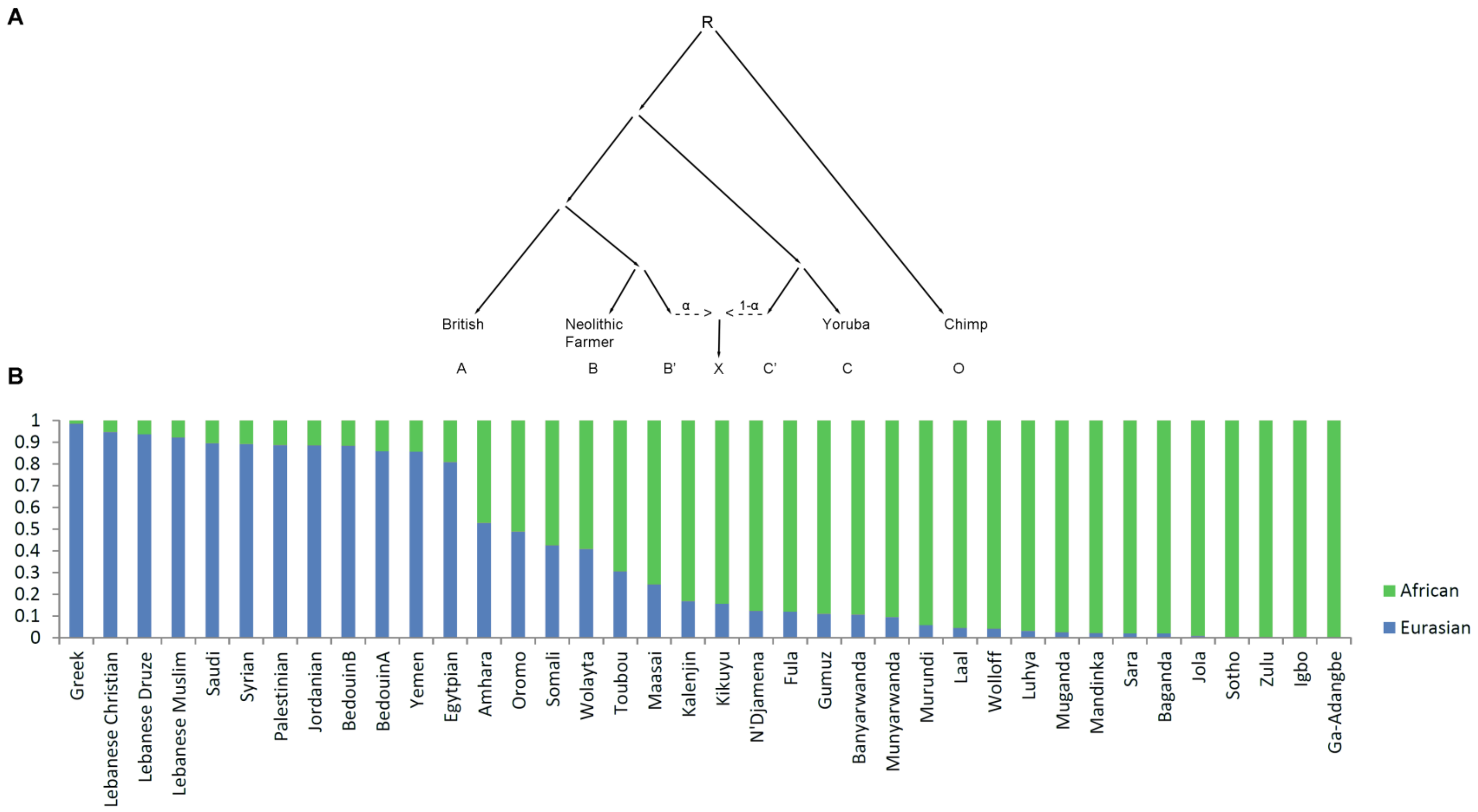

Figure S3. African-Eurasian mixture proportions. We quantify the proportion of admixture using the f_4 ratio, with populations related to European Neolithic farmers and Yoruba as source populations. Eurasian ancestry in Chad ranges from 0.3-2% in Sara to 26-30% in Toubou. A) Population phylogeny used to estimate the African-Eurasian admixture proportions. B) Estimated African-Eurasian ancestry proportions in present-day populations.

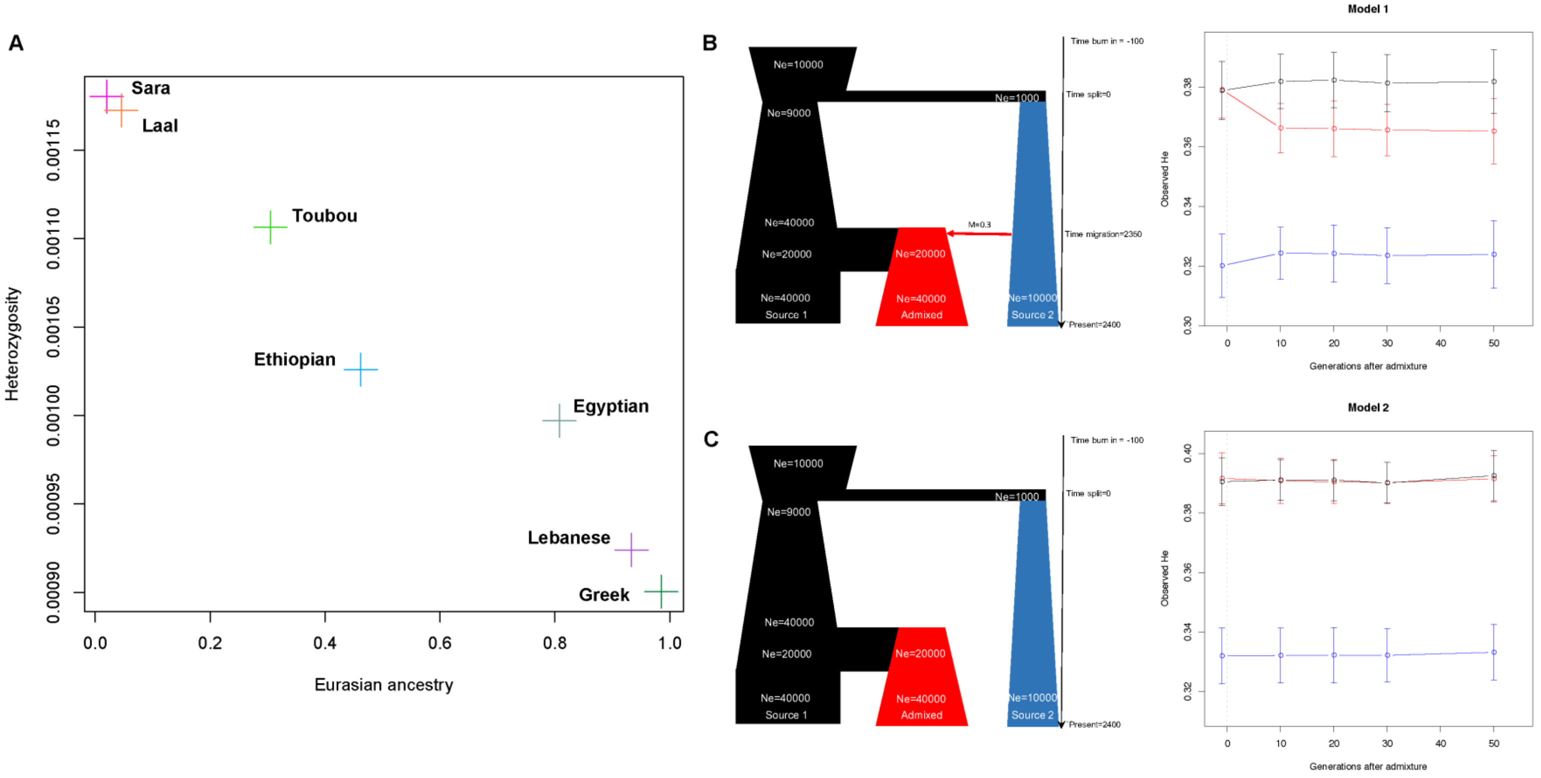

Figure S4. Eurasian admixture consequence for heterozygosity. A) Heterozygosity decline in our whole-genome sequence data is correlated with the proportion of Eurasian ancestry in the population. B) Simulations show that a decrease in heterozygosity is expected after 30% mixture with a population who had a bottleneck in their history, in a model incorporating the decrease in population size in the Eurasians during their exit from Africa (Model 1). C) Structure in the African population without admixture cannot explain the decline in heterozygosity observed in the populations examined (Model 2). Observed He in B and C is mean He estimated across 1000 simulated loci.

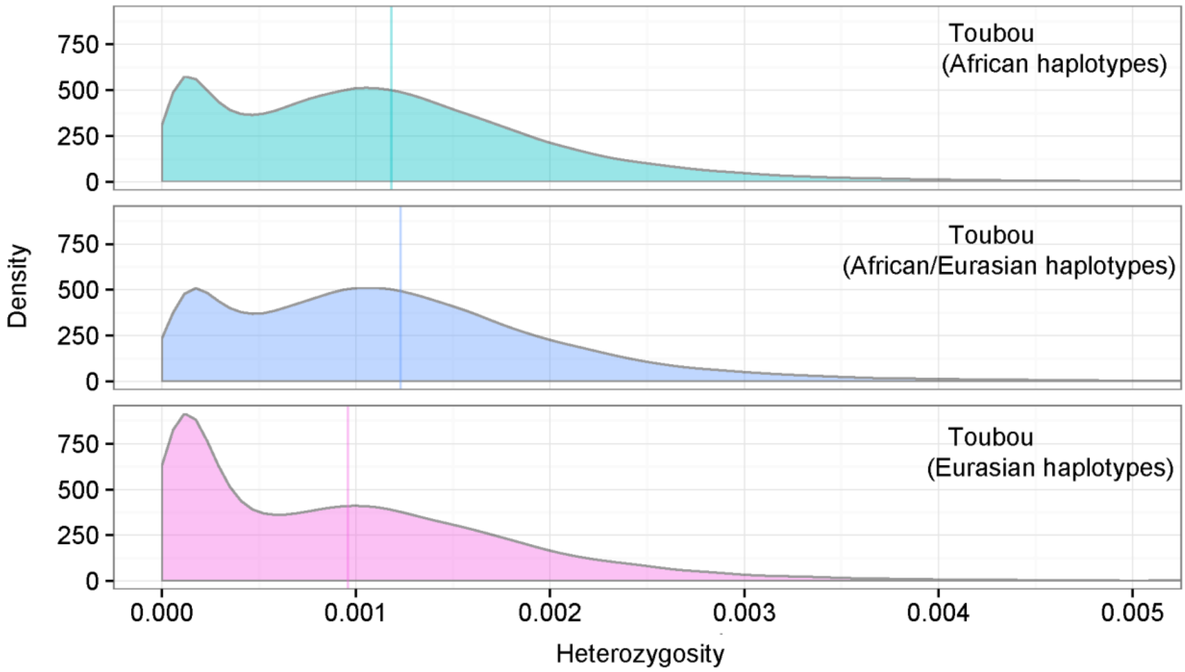

Figure S5. Distribution of heterozygosity in 100kbp windows in whole-genome sequence data from four Toubou individuals. Plots show segments of the Toubou genomes corresponding to different ancestries putatively assigned with PCAdmix. The mean of the distributions is plotted in a vertical line. Admixed African/Eurasian ancestral segments have increased heterozygosity compared with unadmixed African/African segments. However, the Toubou genome segments with complete Eurasian ancestry (Eurasian/Eurasian) have considerable decrease in heterozygosity (~0.96 hets/kbp), leading to the genome-wide decay in heterozygosity pattern observed in Africans with Eurasian ancestry.

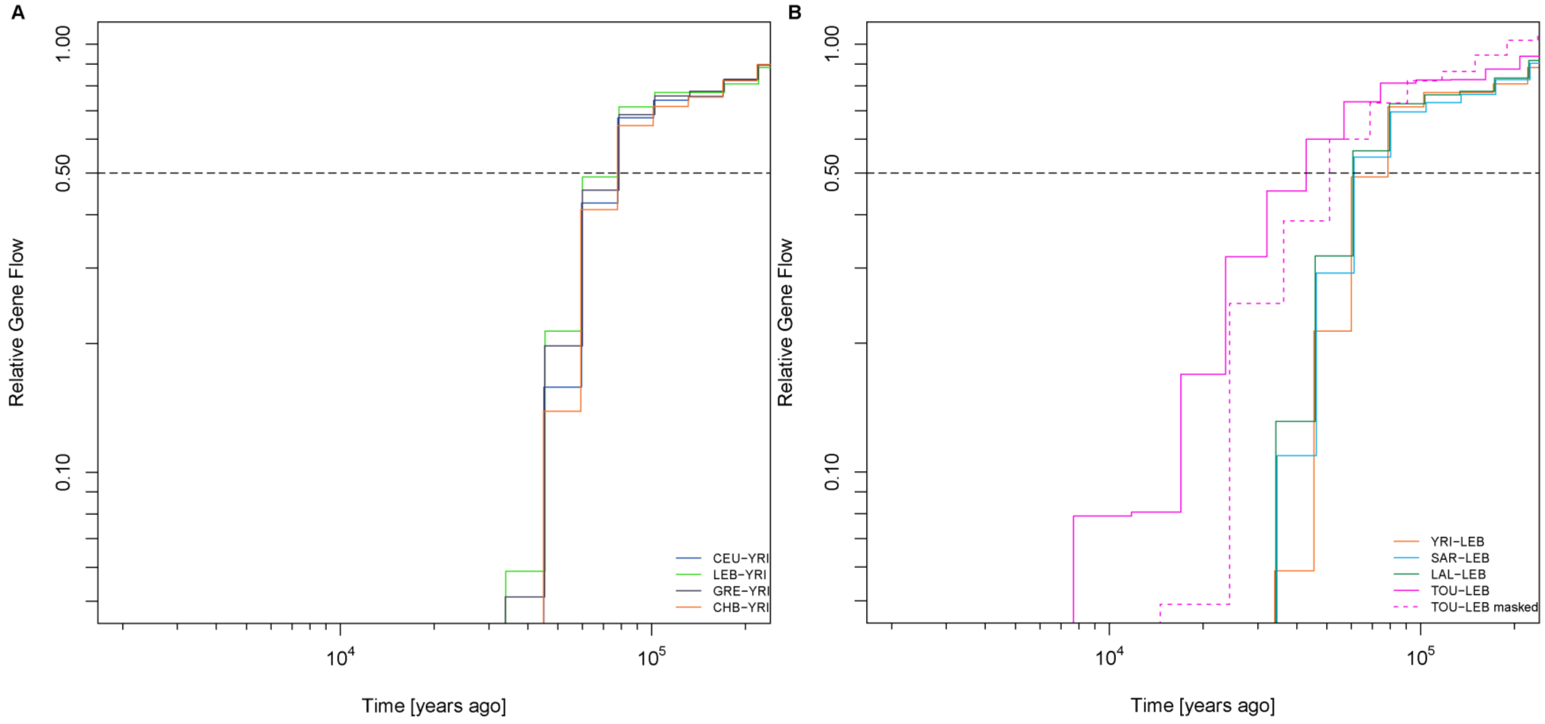

Figure S6. Effect of admixture on the relative cross coalescence rates. Plots show relative gene flow between pairs of populations where a decrease below 50% is assumed to be the time of divergence A) a small proportion of African ancestry <5% in Greek and Lebanese do not bias the inferences from MSMC on their split time from Yoruba, which remains identical to the split time of CEU and CHB from Yoruba. B) A significant proportion of Eurasian ancestry in Toubou (~30%) appears to change their split times from Eurasians towards more recent times compared with other Africans such as Yoruba, Sara, and Laal. Masking part of the Eurasian ancestry in Toubou increases their time of divergence with Eurasians closer to the time of the African-Eurasian split.

Table S1. New populations and data analysed in this paper.

Population	Language family	Location	Country	Region	2.5M array[a]	WGS[a]
Laal	Unclassified	Moyen-Chari	Chad	Africa	26	3
Sara	Nilo-Saharan	Moyen-Chari	Chad	Africa	62	4
Toubou	Nilo-Saharan	Borkou-Ennedi-Tibesti	Chad	Africa	83	4
N'Djamena	Mix	N'Djamena	Chad	Africa	67	
Greek	Indo-European	Athens	Greece	Europe	96[b]	4
Lebanese Christians	Afroasiatic	Beirut, Mount Lebanon, North Lebanon	Lebanon	Near East	52	2
Lebanese Druze	Afroasiatic	Mount Lebanon	Lebanon	Near East	31	
Lebanese Muslims	Afroasiatic	Beirut, Bekaa, North and South Lebanon	Lebanon	Near East	43	2
Yemen	Afroasiatic	Bayda, Dhamar, Marib, Sanaa	Yemen	Near East	20	

[a]Number of individuals
[b]2.5M SNPs extracted from sequence data

Table S2. African-Eurasian admixture time (generations ago) inferred using ALDER.

References	Laal	Sara	Toubou	Amhara
British/Fula	-	155 (±22), z=7.21	49 (±5), z=9.49	90 (±4), z=18.65
British/Ga-Adangbe	190 (±58), z=2.71	146 (±20), z=7.2	48 (±5), z=9.42	90 (±4), z=18.65
British/Igbo	193 (±55), z=2.81	143 (±20), z=7.29	48 (±5), z=9.07	90 (±4), z=18.52
British/Jola	174 (±53), z=2.74	154 (±19), z=8.06	49 (±5), z=9.58	90 (±4), z=17.91
British/Mandinka	196 (±57), z=2.72	151 (±20), z=7.68	49 (±5), z=9.19	91 (±4), z=18.42
British/Wolloff	205 (±48), z=3.13	154 (±19), z=7.96	49 (±5), z=9.27	90 (±4), z=17.97
British/Yoruba	222 (±68), z=2.41	149 (±20), z=7.29	47 (±5), z=9.03	90 (±4), z=18.76
Greek/Fula	-	158 (±20), z=7.91	48 (±5), z=9.27	90 (±4), z=19.53
Greek/Ga-Adangbe	193 (±51), z=3.15	148 (±19), z=7.65	47 (±5), z=9.19	89 (±4), z=19.51
Greek/Igbo	194 (±50), z=3.14	144 (±18), z=7.9	47 (±5), z=8.85	90 (±4), z=19.36
Greek/Jola	180 (±48), z=3.12	160 (±18), z=8.7	48 (±5), z=9.4	90 (±4), z=18.72
Greek/Mandinka	202 (±51), z=3.1	155 (±19), z=7.97	48 (±5), z=9.01	90 (±4), z=19.26
Greek/Wolloff	210 (±44), z=3.55	159 (±19), z=8.18	48 (±5), z=9.05	90 (±4), z=18.78
Greek/Yoruba	222 (±62), z=2.69	153 (±19), z=8.13	46 (±5), z=8.78	90 (±4), z=19.55
Gujarati/Fula	-	187 (±24), z=5.85	46 (±5), z=8.61	89 (±3), z=26.25
Gujarati/Ga-Adangbe	-	164 (±20), z=7.17	46 (±5), z=8.84	89 (±3), z=24.73
Gujarati/Igbo	-	157 (±19), z=6.96	45 (±5), z=8.35	89 (±3), z=24.32
Gujarati/Jola	-	183 (±23), z=6.01	47 (±5), z=9.05	89 (±3), z=23.78
Gujarati/Mandinka	-	175 (±21), z=6.02	47 (±5), z=8.55	90 (±3), z=24.93
Gujarati/Wolloff	-	184 (±22), z=6.36	46 (±5), z=8.82	89 (±3), z=24.1
Gujarati/Yoruba	-	164 (±20), z=6.83	45 (±5), z=8.21	89 (±3), z=24.44
Han/Fula	-	177 (±40), z=3.55	46 (±6), z=7.32	93 (±5), z=14.78
Han/Ga-Adangbe	-	160 (±28), z=5.28	45 (±6), z=7.78	92 (±5), z=15.05
Han/Igbo	-	149 (±26), z=5.73	45 (±6), z=7.26	91 (±5), z=15.06
Han/Jola	-	165 (±25), z=5.54	47 (±6), z=7.72	93 (±5), z=14.4
Han/Mandinka	-	174 (±28), z=4.83	46 (±6), z=7.38	93 (±5), z=15.12
Han/Wolloff	-	181 (±31), z=4.71	46 (±6), z=7.67	93 (±5), z=14.24
Han/Yoruba	-	162 (±33), z=4.73	44 (±6), z=7.25	92 (±5), z=14.99
Lebanese_Christian/Fula	-	158 (±22), z=7.2	48 (±5), z=8.71	91 (±4), z=18.67
Lebanese_Christian/Ga-Adangbe	199 (±54), z=2.98	148 (±19), z=7.77	47 (±5), z=8.64	90 (±4), z=18.69
Lebanese_Christian/Igbo	202 (±54), z=2.94	145 (±19), z=7.84	46 (±6), z=8.34	91 (±4), z=18.64
Lebanese_Christian/Jola	191 (±54), z=2.78	160 (±20), z=7.77	48 (±5), z=8.86	91 (±4), z=18.05
Lebanese_Christian/Mandinka	215 (±56), z=2.87	156 (±21), z=7.1	48 (±6), z=8.48	91 (±4), z=18.32
Lebanese_Christian/Wolloff	218 (±46), z=3.41	160 (±20), z=7.98	47 (±6), z=8.52	91 (±4), z=18
Lebanese_Christian/Yoruba	226 (±63), z=2.66	153 (±19), z=8.23	46 (±6), z=8.25	91 (±4), z=18.75
Lebanese_Druze/Fula	-	164 (±25), z=6.52	47 (±6), z=8.55	89 (±3), z=20.73
Lebanese_Druze/Ga-Adangbe	206 (±57), z=2.88	149 (±20), z=7.33	46 (±5), z=8.5	89 (±4), z=20.11
Lebanese_Druze/Igbo	210 (±56), z=2.9	146 (±18), z=7.92	46 (±6), z=8.22	89 (±4), z=20.05
Lebanese_Druze/Jola	195 (±52), z=2.92	164 (±21), z=7.61	48 (±5), z=8.7	90 (±4), z=19.42
Lebanese_Druze/Mandinka	219 (±56), z=2.88	160 (±23), z=7	47 (±6), z=8.31	90 (±4), z=19.76
Lebanese_Druze/Wolloff	221 (±46), z=3.43	164 (±22), z=7.45	47 (±6), z=8.37	90 (±4), z=19.29
Lebanese_Druze/Yoruba	232 (±68), z=2.5	154 (±19), z=8.09	46 (±6), z=8.12	90 (±3), z=20.29
Lebanese_Muslim/Fula	-	161 (±23), z=7.14	48 (±5), z=9.11	90 (±4), z=20.71
Lebanese_Muslim/Ga-Adangbe	208 (±54), z=3.05	150 (±20), z=7.43	47 (±5), z=9.02	89 (±4), z=20.24
Lebanese_Muslim/Igbo	211 (±54), z=3.06	147 (±19), z=7.7	47 (±5), z=8.68	89 (±4), z=20.02
Lebanese_Muslim/Jola	196 (±52), z=2.99	163 (±20), z=8.21	48 (±5), z=9.2	90 (±4), z=19.62
Lebanese_Muslim/Mandinka	223 (±54), z=3.06	158 (±21), z=7.36	48 (±5), z=8.79	90 (±4), z=19.91
Lebanese_Muslim/Wolloff	225 (±44), z=3.66	163 (±21), z=7.73	48 (±5), z=8.83	90 (±4), z=19.46
Lebanese_Muslim/Yoruba	238 (±64), z=2.74	154 (±20), z=7.67	46 (±5), z=8.53	90 (±4), z=20.27
Yemen/Fula	-	158 (±27), z=5.75	48 (±6), z=8	89 (±3), z=23.03
Yemen/Ga-Adangbe	189 (±45), z=3.38	145 (±21), z=6.85	46 (±6), z=7.94	89 (±3), z=22.21
Yemen/Igbo	195 (±47), z=3.26	145 (±20), z=7.25	46 (±6), z=7.74	89 (±3), z=22.21
Yemen/Jola	182 (±46), z=3.13	160 (±22), z=7.18	48 (±6), z=8.2	90 (±4), z=21.63
Yemen/Mandinka	202 (±48), z=3.22	155 (±24), z=6.39	48 (±6), z=7.88	90 (±4), z=21.9
Yemen/Wolloff	212 (±42), z=3.62	160 (±23), z=6.93	47 (±6), z=7.83	90 (±4), z=21.71
Yemen/Yoruba	212 (±55), z=2.93	150 (±21), z=7.11	46 (±6), z=7.59	89 (±3), z=22.58

Table S3. MALDER results (generations ago) allowing multiple admixture events.

	First event		Second event	
**Population**	**Reference**	**Time (sd), z-score**	**Reference**	**Time (sd), z-score**
Amhara	Laal/Greek	89(±4), z=26.28	-	-
Laal	Igbo/British	206(±42), z=4.86	-	-
Sara	Igbo/Greek	150(±15), z=9.81		
Toubou	Laal/British	109(±10), z=10.65	Laal/Greek	7(±2), z=5.12

Table S4. Y chromosomal haplogroup frequencies. Haplogroups were defined based on 636 Y-chromosomal SNPs overlapping with ISOGG markers (July 2016).

Haplogroup	Nigeria	Chad				Ethiopia					Kenya		Egypt	Lebanon			Yemen
	Yoruba	Laal	Sara	N'Djamena	Toubou	Amhara	Oromo	Somali	Wolayta	Gumuz	Luhya	Maasai	Egyptian	Christian	Druze	Muslim	Yemeni
A1b1b2b-M63	0	0	0.02	0.13	0	0.30	0.17	0	0.43	0.59	0	0.06	0.01	0	0	0	0
B-M181	0	0.54	0.10	0	0	0	0	0	0	0	0	0	0	0	0	0	0
B2-M247	0	0	0	0	0	0	0	0	0	0.41	0.02	0.06	0	0	0	0	0
B2a-M150	0	0	0.05	0.02	0	0	0	0	0	0	0.04	0	0	0	0	0	0
B2a1a1a1-M109	0	0	0.02	0.04	0	0	0	0	0	0	0	0	0	0	0	0	0
B2b-M112	0	0	0.02	0.02	0	0	0	0	0	0	0	0	0.01	0	0	0	0
E-M96	0.02	0	0	0	0	0	0	0	0.05	0	0.08	0.24	0	0	0	0	0
E1a-M132	0.07	0	0.02	0.05	0	0	0	0	0	0	0	0	0	0	0	0.02	0
E1b1a-V38	0	0	0	0	0	0.04	0	0	0.10	0	0	0	0	0	0	0	0
E1b1a1a1-P88	0	0	0	0	0	0	0	0	0	0	0	0	0.01	0	0	0	0
E1b1a1a1a-M58	0	0	0.02	0.02	0	0	0	0	0	0	0	0	0	0	0	0	0
E1b1a1a1b-M10	0	0.04	0.02	0	0.03	0	0	0	0	0	0	0	0	0	0	0	0
E1b1a1a1c-L458	0	0	0	0	0	0	0	0	0	0	0	0	0.01	0	0	0	0
E1b1a1a1c1a-P86	0.44	0.04	0.23	0.11	0.01	0	0	0	0	0	0.64	0.06	0	0	0	0	0
E1b1a1a1c1b-L516	0	0.15	0.18	0.07	0.01	0	0	0	0	0	0	0	0	0	0	0	0
E1b1a1a1d1-P277	0.47	0	0.07	0	0	0	0	0	0	0	0.14	0.12	0.02	0	0	0	0.05
E1b1b-PF1942	0	0	0	0.02	0.04	0.22	0.22	0	0.19	0	0.08	0.47	0	0	0	0.05	0.05
E1b1b1a1-PF2186	0	0	0.07	0.15	0.21	0.15	0.22	0.88	0.05	0	0	0	0.34	0.04	0.03	0	0
E1b1b1b1a1-M183	0	0	0	0.02	0.03	0	0	0	0	0	0	0	0.05	0	0.16	0.02	0
E1b1b1b2a-M123	0	0	0	0	0	0.04	0.06	0	0.19	0	0	0	0.09	0.02	0	0	0.20
E2-M75	0	0	0.02	0	0	0	0	0	0	0	0	0	0.01	0	0	0	0
G1-M285	0	0	0	0	0	0	0	0	0	0	0	0	0	0.06	0	0.02	0
G2-PF3138	0	0	0	0	0	0	0	0	0	0	0	0	0	0.08	0	0.02	0
G2a-PF3112	0	0	0	0	0	0	0	0	0	0	0	0	0	0	0.03	0.02	0.05
G2a2b-S126	0	0	0	0	0	0	0	0	0	0	0	0	0.05	0.06	0	0.12	0
G2b1-M377	0	0	0	0	0	0	0	0	0	0	0	0	0.01	0.02	0	0.02	0
H1-M69	0	0	0	0	0	0	0	0	0	0	0	0	0	0	0.06	0	0
I2-S329	0	0	0	0	0	0	0	0	0	0	0	0	0	0.02	0	0	0
J1-PF4782	0	0	0	0.13	0.01	0.11	0.33	0	0	0	0	0	0.20	0.08	0	0.10	0.60
J2-PF4908	0	0	0	0	0	0	0	0	0	0	0	0	0.01	0.06	0	0	0
J2a1-L26	0	0	0	0	0	0.07	0	0.06	0	0	0	0	0.05	0.17	0.29	0.29	0
J2b-PF4939	0	0	0	0	0	0.04	0	0	0	0	0	0	0	0	0	0	0
L1-M22	0	0	0	0	0	0	0	0	0	0	0	0	0	0.08	0.03	0	0
L1a1-M27	0	0	0	0	0	0	0	0	0	0	0	0	0.01	0	0.10	0.07	0
L1b1-M349	0	0	0	0	0	0	0	0	0	0	0	0	0	0.04	0	0	0
Q-M242	0	0	0	0	0	0	0	0	0	0	0	0	0	0.02	0	0.02	0
R1a1a1-M417	0	0	0	0	0	0	0	0	0	0	0	0	0.01	0	0	0	0
R1b1a-L761	0	0.23	0.20	0.15	0.34	0	0	0	0	0	0	0	0.03	0	0.03	0	0
R1b1a1a-P297	0	0	0	0	0	0	0	0	0	0	0	0	0.02	0	0	0	0
R1b1a1a2-L500	0	0	0	0	0	0	0	0	0	0	0	0	0.02	0.15	0.16	0	0
R1b1a1a2a1a-S128	0	0	0	0	0	0	0	0	0	0	0	0	0.01	0.04	0.03	0.05	0
R2a-P267	0	0	0	0	0	0	0	0	0	0	0	0	0	0	0	0.02	0
T-M272	0	0	0	0	0	0	0	0	0	0	0	0	0	0.02	0	0	0
T1a-PF5662	0	0	0	0.09	0.31	0.04	0	0.06	0	0	0	0	0.02	0.06	0.06	0.14	0.05
**No. of samples**	**43**	**26**	**61**	**55**	**71**	**27**	**18**	**16**	**21**	**17**	**50**	**17**	**99**	**52**	**31**	**42**	**20**

Table S5. Admixture f_3 statistics *(Test; Ref1, Yoruba)* where Ref1 is a present-day non sub-Saharan African population.

		Toubou			Amhara		
**Reference 1**	**Region**	**f3 values**	**S.E.**	**z-score**	**f3 values**	**S.E.**	**z-score**
Sardinian	Europe	-0.014559	0.000365	-39.84	-0.026489	0.000366	-72.309
Bergamo	Europe	-0.013812	0.000363	-38.085	-0.02557	0.00037	-69.149
Cypriot	Near East	-0.013104	0.000369	-35.502	-0.025539	0.000365	-70.001
Spanish_North	Europe	-0.014066	0.000396	-35.564	-0.025537	0.000425	-60.125
Basque	Europe	-0.013985	0.000369	-37.924	-0.025435	0.000373	-68.149
Lebanese_Christian	Near East	-0.012978	0.000341	-38.025	-0.025404	0.000315	-80.534
Tuscan	Europe	-0.013329	0.000384	-34.665	-0.025246	0.000401	-62.954
Greek	Europe	-0.01327	0.000352	-37.676	-0.025239	0.000333	-75.683
Georgian_Jew	Near East	-0.012718	0.000383	-33.196	-0.025085	0.000416	-60.311
Albanian	Europe	-0.013315	0.000397	-33.524	-0.025003	0.00041	-60.956
French_South	Europe	-0.013644	0.000384	-35.574	-0.024881	0.000404	-61.578
Iraqi_Jew	Near East	-0.01264	0.000384	-32.903	-0.02484	0.000403	-61.697
Yemenite_Jew	Near East	-0.012205	0.000367	-33.294	-0.024829	0.000389	-63.876
Armenian	Near East	-0.012636	0.000372	-34.011	-0.024781	0.000378	-65.59
Bulgarian	Europe	-0.013256	0.000377	-35.181	-0.024739	0.000383	-64.622
Italian_South	Europe	-0.013833	0.000537	-25.758	-0.024663	0.000632	-39.018
French	Europe	-0.013391	0.000363	-36.905	-0.024597	0.000362	-68
Croatian	Europe	-0.013172	0.00038	-34.623	-0.024539	0.000416	-59.045
English	Europe	-0.013171	0.000382	-34.466	-0.024325	0.000397	-61.341
Czech	Europe	-0.013014	0.000378	-34.396	-0.024285	0.000386	-62.953
Orcadian	Europe	-0.013003	0.000377	-34.53	-0.024245	0.000387	-62.609
British	Europe	-0.013094	0.000358	-36.543	-0.024234	0.00036	-67.368
Spanish	Europe	-0.013355	0.000351	-38.02	-0.024169	0.000342	-70.719
Georgian	Caucasus	-0.012282	0.000378	-32.491	-0.024142	0.000381	-63.354
Hungarian	Europe	-0.012911	0.000361	-35.785	-0.024109	0.000364	-66.185
Abkhasian	Caucasus	-0.012216	0.000378	-32.332	-0.024098	0.000399	-60.468
Saudi	Near East	-0.011961	0.000366	-32.66	-0.024089	0.000384	-62.666
Druze	Near East	-0.012417	0.000345	-36.019	-0.02407	0.000338	-71.197
Scottish	Europe	-0.013019	0.000413	-31.516	-0.024065	0.000451	-53.381
Icelandic	Europe	-0.013039	0.000384	-33.957	-0.024047	0.000396	-60.76
Norwegian	Europe	-0.013007	0.000381	-34.165	-0.024037	0.000398	-60.465
Lebanese_Druze	Near East	-0.012339	0.00035	-35.285	-0.024037	0.000336	-71.614
BedouinB	Near East	-0.012134	0.000339	-35.839	-0.024031	0.00034	-70.607
Sicilian	Europe	-0.012809	0.000371	-34.547	-0.023986	0.000373	-64.363
Lithuanian	Europe	-0.012663	0.000384	-33.003	-0.023674	0.000395	-59.922
Chechen	Caucasus	-0.012014	0.000371	-32.374	-0.023531	0.000388	-60.657
Ukrainian	Europe	-0.012641	0.000381	-33.179	-0.02347	0.0004	-58.668
Belarusian	Europe	-0.012575	0.000364	-34.508	-0.023319	0.000377	-61.854
Turkish	Near East	-0.012011	0.000339	-35.456	-0.023315	0.000324	-71.941
Adygei	Caucasus	-0.011843	0.000365	-32.418	-0.023298	0.000361	-64.504
Estonian	Europe	-0.012462	0.000374	-33.356	-0.022913	0.000393	-58.298
Lezgin	Caucasus	-0.011553	0.000369	-31.311	-0.022832	0.000374	-61.051
North_Ossetian	Caucasus	-0.011865	0.000368	-32.255	-0.022811	0.000377	-60.526
Lebanese_Muslim	Near East	-0.011667	0.000334	-34.957	-0.022727	0.000316	-71.857
Kumyk	Caucasus	-0.011635	0.000374	-31.073	-0.02263	0.00038	-59.601
Balkar	Caucasus	-0.011489	0.000369	-31.104	-0.022566	0.000386	-58.497
Russian	Europe	-0.012159	0.000361	-33.643	-0.022544	0.000369	-61.132
Finnish	Europe	-0.012297	0.000385	-31.955	-0.022498	0.000419	-53.694
Iranian	Near East	-0.011122	0.000362	-30.736	-0.022061	0.000381	-57.84
Lebanese	Near East	-0.011236	0.000349	-32.225	-0.021939	0.000351	-62.511
Yemeni	Near East	-0.010514	0.000321	-32.709	-0.02142	0.000299	-71.645
Syrian	Near East	-0.010962	0.000357	-30.686	-0.020863	0.000387	-53.905
Palestinian	Near East	-0.010886	0.00033	-33.034	-0.020802	0.00031	-67.094
Jordanian	Near East	-0.010636	0.000345	-30.841	-0.020252	0.000369	-54.911
Nogai	Caucasus	-0.010308	0.000371	-27.809	-0.019833	0.00039	-50.88
Tajik_Pomiri	central south asia	-0.009977	0.000367	-27.185	-0.019561	0.000385	-50.805
BedouinA	Near East	-0.010237	0.00032	-31.946	-0.018794	0.000312	-60.297
Balochi	central south asia	-0.009303	0.000352	-26.446	-0.018555	0.000361	-51.426
Brahui	central south asia	-0.009232	0.000348	-26.496	-0.018242	0.000355	-51.453
Kalash	central south asia	-0.009076	0.000389	-23.338	-0.017955	0.000422	-42.503
Pathan	central south asia	-0.009038	0.000348	-25.992	-0.017829	0.000361	-49.385
Cochin_Jew	Near East	-0.009278	0.00038	-24.416	-0.017737	0.000434	-40.889
Makrani	central south asia	-0.008611	0.000341	-25.229	-0.017194	0.000354	-48.515
Sindhi	central south asia	-0.008649	0.000342	-25.254	-0.017021	0.000372	-45.77
Burusho	central south asia	-0.008692	0.000343	-25.347	-0.016624	0.000358	-46.447
Uzbek	East Asia	-0.008837	0.000361	-24.499	-0.016574	0.000405	-40.876
GujaratiA	central south asia	-0.008589	0.000369	-23.264	-0.016445	0.000434	-37.884

Table S5 (continued). Admixture f_3 statistics *(Test; Ref1, Yoruba)* where Ref1 is a present-day non sub-Saharan African population.

		Toubou			Amhara		
**Reference 1**	**Region**	**f3 values**	**S.E.**	**z-score**	**f3 values**	**S.E.**	**z-score**
GujaratiB	central south asia	-0.008189	0.00039	-20.987	-0.015728	0.000443	-35.483
EgyptianA	North Africa	-0.009115	0.000306	-29.79	-0.015626	0.000312	-50.052
Mansi	East Asia	-0.008356	0.000399	-20.937	-0.015385	0.000469	-32.797
EgytpianB	North Africa	-0.008726	0.000283	-30.818	-0.015107	0.000246	-61.381
Gujarati	central south asia	-0.007651	0.000338	-22.632	-0.014868	0.000364	-40.859
Hazara	central south asia	-0.007893	0.000366	-21.581	-0.014858	0.000413	-36.017
Uygur	central south asia	-0.007818	0.000364	-21.456	-0.014844	0.000421	-35.284
GujaratiC	central south asia	-0.007571	0.000395	-19.145	-0.014788	0.000457	-32.388
Punjabi	central south asia	-0.007362	0.000359	-20.532	-0.014278	0.000417	-34.274
GujaratiD	central south asia	-0.00739	0.000385	-19.186	-0.014268	0.000452	-31.581
Tubalar	East Asia	-0.007636	0.000387	-19.752	-0.013625	0.000457	-29.83
Even	East Asia	-0.007523	0.000391	-19.259	-0.01355	0.00047	-28.834
Selkup	East Asia	-0.007504	0.000393	-19.075	-0.013386	0.000481	-27.814
Bengali	central south asia	-0.007008	0.000385	-18.193	-0.013124	0.000438	-29.956
Kyrgyz	central south asia	-0.007343	0.000384	-19.146	-0.013113	0.000473	-27.721
Altaian	East Asia	-0.006764	0.000395	-17.113	-0.012378	0.000497	-24.928
Quechua	Native American	-0.006357	0.000454	-13.994	-0.01162	0.000573	-20.286
Bolivian	Native American	-0.006456	0.000433	-14.926	-0.011307	0.000571	-19.802
Kalmyk	East Asia	-0.006183	0.000401	-15.414	-0.011269	0.000494	-22.832
Yukagir	East Asia	-0.006395	0.000392	-16.317	-0.011107	0.000493	-22.542
Surui	Native American	-0.006167	0.000504	-12.229	-0.011103	0.000677	-16.406
Tunisian	North Africa	-0.008142	0.000331	-24.62	-0.011078	0.000383	-28.931
Tuvinian	East Asia	-0.006255	0.000404	-15.487	-0.011065	0.000508	-21.785
Zapotec	Native American	-0.00641	0.000435	-14.743	-0.010943	0.000574	-19.073
Pima	Native American	-0.005948	0.000461	-12.895	-0.010651	0.000596	-17.865
Yakut	East Asia	-0.006216	0.000403	-15.434	-0.010639	0.000508	-20.952
Karitiana	Native American	-0.006142	0.000479	-12.813	-0.010628	0.000645	-16.486
Mayan	Native American	-0.006023	0.000421	-14.317	-0.010598	0.000559	-18.964
Mixe	Native American	-0.00613	0.000457	-13.404	-0.010559	0.000612	-17.253
Dolgan	East Asia	-0.006012	0.000445	-13.516	-0.010505	0.000575	-18.266
Mixtec	Native American	-0.005738	0.000439	-13.068	-0.009851	0.000588	-16.76
Thule	East Asia	-0.006153	0.001678	-3.667	-0.009729	0.002297	-4.236
Piapoco	Native American	-0.005517	0.000458	-12.037	-0.009676	0.000642	-15.078
Kusunda	East Asia	-0.005242	0.000409	-12.822	-0.0093	0.000512	-18.163
Itelmen	East Asia	-0.005389	0.000449	-11.999	-0.009144	0.000606	-15.093
Oroqen	East Asia	-0.005094	0.000422	-12.058	-0.009079	0.00055	-16.502
Mongola	East Asia	-0.005126	0.000432	-11.865	-0.009008	0.000563	-16.009
Mozabite	North Africa	-0.007802	0.000291	-26.781	-0.008966	0.000344	-26.057
Nganasan	East Asia	-0.005211	0.000445	-11.72	-0.008881	0.000592	-14.992
Koryak	East Asia	-0.005165	0.000435	-11.885	-0.008826	0.000573	-15.406
Xibo	East Asia	-0.005129	0.000429	-11.946	-0.008775	0.000549	-15.987
Algerian	North Africa	-0.007087	0.000352	-20.136	-0.008766	0.000457	-19.17
Nivkh	East Asia	-0.004748	0.00049	-9.687	-0.008761	0.000652	-13.43
Daur	East Asia	-0.005122	0.000415	-12.353	-0.008711	0.000533	-16.333
Tu	East Asia	-0.005086	0.000412	-12.348	-0.00865	0.000527	-16.421
Han_NChina	East Asia	-0.005099	0.00042	-12.15	-0.008563	0.000537	-15.944
Thai	East Asia	-0.005003	0.000412	-12.153	-0.00853	0.000519	-16.439
Hezhen	East Asia	-0.005014	0.000413	-12.135	-0.008519	0.000557	-15.302
Ulchi	East Asia	-0.004847	0.000415	-11.692	-0.008374	0.000546	-15.35
Korean	East Asia	-0.004918	0.000442	-11.136	-0.008306	0.000577	-14.404
Cambodian	East Asia	-0.004927	0.000411	-11.995	-0.00829	0.000527	-15.731
Tujia	East Asia	-0.004867	0.000426	-11.429	-0.008161	0.000538	-15.18
Miao	East Asia	-0.004718	0.000432	-10.913	-0.008154	0.000554	-14.711
Han	East Asia	-0.00476	0.000412	-11.563	-0.008064	0.000526	-15.327
Japanese	East Asia	-0.004718	0.000419	-11.26	-0.008058	0.000538	-14.972
Dai	East Asia	-0.004716	0.000426	-11.063	-0.007908	0.000541	-14.621
Yi	East Asia	-0.004648	0.00042	-11.063	-0.007863	0.000532	-14.772
Atayal	East Asia	-0.004587	0.000454	-10.101	-0.007837	0.000586	-13.365
She	East Asia	-0.004784	0.000432	-11.066	-0.007781	0.000566	-13.751
Ami	East Asia	-0.00458	0.000443	-10.331	-0.007733	0.000566	-13.663
Naxi	East Asia	-0.004585	0.000422	-10.856	-0.007716	0.000532	-14.493
Lahu	East Asia	-0.004561	0.000433	-10.533	-0.007672	0.000543	-14.131
Kinh	East Asia	-0.004431	0.000434	-10.201	-0.007414	0.000549	-13.5
Saharawi	North Africa	-0.007584	0.000339	-22.367	-0.00733	0.000448	-16.35
Botocudo	Native American	-0.003583	0.000605	-5.927	-0.005898	0.000835	-7.061
Bougainville	Oceanian	-0.00311	0.000444	-6.997	-0.005312	0.000658	-8.074
Australian	Oceanian	-0.002694	0.000506	-5.321	-0.004905	0.000753	-6.517
Papuan	Oceanian	-0.00236	0.000474	-4.978	-0.004038	0.000691	-5.84

Table S6. Admixture f_3 statistics *(Test; Ref1, Yoruba)* where Ref1 is an ancient population.

		Toubou			Amhara		
**Reference 1**	**Period and region**	**f3 values**	**S.E.**	**z-score**	**f3 values**	**S.E.**	**z-score**
LBK_EN	Early Neolithic European	-0.014918	0.000428	-34.818	-0.028068	0.000456	-61.539
Spain_EN	Early Neolithic European	-0.015126	0.00046	-32.861	-0.027009	0.000526	-51.35
HungaryGamba_EN	Early Neolithic European	-0.015156	0.000475	-31.934	-0.0283	0.000523	-54.072
Spain_MN	Middle Neolithic European	-0.014369	0.000486	-29.542	-0.025615	0.000574	-44.601
Unetice_EBA	Bronze Age Euopean	-0.012449	0.000441	-28.227	-0.023412	0.000497	-47.153
Bell_Beaker_LN	Late Neolithic European	-0.012543	0.000447	-28.031	-0.023304	0.000535	-43.551
Stuttgart	Early Neolithic European	-0.014762	0.000528	-27.958	-0.027612	0.000668	-41.362
Motala_HG	Hunter-gatherer European	-0.011612	0.000447	-25.953	-0.020525	0.000567	-36.22
Corded_Ware_LN	Late Neolithic European	-0.011707	0.000492	-23.795	-0.022564	0.00058	-38.892
Yamnaya	Bronze Age Eurasian	-0.010322	0.000439	-23.528	-0.020166	0.000499	-40.401
HungaryGamba_BA	Bronze Age Euopean	-0.012701	0.000575	-22.093	-0.023871	0.000696	-34.319
Iceman	Copper Age European	-0.01358	0.000621	-21.884	-0.025223	0.0008	-31.542
Loschbour	Hunter-gatherer European	-0.012448	0.000571	-21.783	-0.021552	0.000739	-29.175
SwedenSkoglund_NHG	Hunter-gatherer European	-0.012629	0.00059	-21.4	-0.02203	0.000786	-28.013
Esperstedt_MN	Middle Neolithic European	-0.014963	0.000713	-20.976	-0.028653	0.000892	-32.117
BenzigerodeHeimburg_LN	Late Neolithic European	-0.011048	0.000538	-20.541	-0.021253	0.000677	-31.405
Halberstadt_LBA	Bronze Age Euopean	-0.012164	0.000602	-20.191	-0.023085	0.000794	-29.065
Baalberge_MN	Middle Neolithic European	-0.014442	0.000733	-19.707	-0.02697	0.000848	-31.817
Alberstedt_LN	Late Neolithic European	-0.012738	0.00066	-19.304	-0.023392	0.000813	-28.776
SwedenSkoglund_MN	Middle Neolithic European	-0.013857	0.000723	-19.157	-0.025893	0.000931	-27.803
HungaryGamba_CA	Copper Age European	-0.014009	0.000757	-18.514	-0.026447	0.000944	-28.006
HungaryGamba_HG	Hunter-gatherer European	-0.012531	0.000684	-18.317	-0.02242	0.000904	-24.795
LaBrana1	Hunter-gatherer European	-0.011231	0.00062	-18.101	-0.01938	0.000827	-23.437
HungaryGamba_IA	Iron Age European	-0.012442	0.000695	-17.91	-0.023381	0.000936	-24.969
Samara_HG	Hunter-gatherer European	-0.011103	0.000722	-15.389	-0.018467	0.000969	-19.06
Starcevo_EN	Early Neolithic European	-0.015168	0.000993	-15.279	-0.028631	0.001227	-23.326
Karelia_HG	Hunter-gatherer European	-0.009448	0.000646	-14.621	-0.01781	0.000804	-22.161
Kostenki14	Paleolithic Eurasian	-0.008015	0.000688	-11.651	-0.014527	0.00089	-16.315
MA1	Paleolithic Eurasian	-0.007973	0.000692	-11.515	-0.015407	0.000933	-16.508
Karsdorf_LN	Late Neolithic European	-0.012201	0.001301	-9.375	-0.022034	0.001615	-13.643
Ust_Ishim	Paleolithic Eurasian	-0.002688	0.000567	-4.745	-0.003908	0.000806	-4.846
