# Supplementary material for: Chad Genetic Diversity Reveals an African History Marked by Multiple Holocene Eurasian Migrations
Source: Am J Hum Genet. 2016 Nov 23;99(6):1316–24. doi: 10.1016/j.ajhg.2016.10.012 (PMC5142112; doi:10.1016/j.ajhg.2016.10.012)
Supplement: Document S2. Article plus Supplemental Data [file mmc2.pdf]

Chad Genetic Diversity Reveals an African History Marked by Multiple Holocene Eurasian Migrations

Marc Haber,[1,*] Massimo Mezzavilla,[1,2] Anders Bergström,[1] Javier Prado-Martinez,[1] Pille Hallast,[1,3] Riyadh Saif-Ali,[4] Molham Al-Habori,[4] George Dedoussis,[5] Eleftheria Zeggini,[1] Jason Blue-Smith,[6,10] R. Spencer Wells,[7] Yali Xue,[1] Pierre A. Zalloua,[8,9] and Chris Tyler-Smith[1,*]

Understanding human genetic diversity in Africa is important for interpreting the evolution of all humans, yet vast regions in Africa, such as Chad, remain genetically poorly investigated. Here, we use genotype data from 480 samples from Chad, the Near East, and southern Europe, as well as whole-genome sequencing from 19 of them, to show that many populations today derive their genomes from ancient African-Eurasian admixtures. We found evidence of early Eurasian backflow to Africa in people speaking the unclassified isolate Laal language in southern Chad and estimate from linkage-disequilibrium decay that this occurred 4,750–7,200 years ago. It brought to Africa a Y chromosome lineage (R1b-V88) whose closest relatives are widespread in present-day Eurasia; we estimate from sequence data that the Chad R1b-V88 Y chromosomes coalesced 5,700–7,300 years ago. This migration could thus have originated among Near Eastern farmers during the African Humid Period. We also found that the previously documented Eurasian backflow into Africa, which occurred ~3,000 years ago and was thought to be mostly limited to East Africa, had a more westward impact affecting populations in northern Chad, such as the Toubou, who have 20%–30% Eurasian ancestry today. We observed a decline in heterozygosity in admixed Africans and found that the Eurasian admixture can bias inferences on their coalescent history and confound genetic signals from adaptation and archaic introgression.

Introduction

African genetic diversity is still incompletely understood, and vast regions in Africa remain genetically undocumented. Chad, for example, makes up ~5% of Africa's surface area, and its central location, connecting sub-Saharan Africa with North and East Africa, positions it to play an important role as a crossroad or barrier to human migrations. However, Chad has been little studied at a whole-genome level, and its position within African genetic diversity is not well known. With 200 ethnic groups and more than 120 indigenous languages and dialects, Chad has extensive ethnolinguistic diversity.[1] It has been suggested that this diversity can be attributed to Lake Chad, which has attracted human populations to its fertile surroundings since prehistoric times, especially after the progressive desiccation of the Sahara starting ~7,000 years ago (ya).[2,3]

Important questions about Africa's ethnic diversity are the relationships among the different groups and the relationships between cultural groups and existing genetic structures. In the present study, we analyzed four Chadian populations with different ethnicities, languages, and modes of subsistence. Our samples are likely to capture recent genetic signals of migration and mixing and also have the potential to show ancestral genomic relationships that are shared among Chadians and other populations. An additional major question relates to the prehistoric Eurasian migrations to Africa: what was the extent of these migrations, how have they affected African genetic diversity, and what present-day populations harbor genetic signals from the ancient migrating Eurasians? We have previously reported evidence of gene flow from the Near East to East Africa ~3,000 ya, as well as subsequent selection in Ethiopians on non-African-derived alleles related to light skin pigmentation.[4] A recent attempt to quantify the extent of such backflow into Africa more generally, by using ancient DNA (aDNA), suggested that the impact of the Eurasian migration was mostly limited to East Africa.[5] However, previous studies using mitochondrial DNA and the Y chromosome in populations from the Chad Basin found some with an East African[6] or Mediterranean and Eurasian influence,[7,8] and analysis based on genome-wide data[9] found a non-African component (suggested to be from East Africa) in central Sahelian populations. Thus, studying diverse Chadian populations on a whole-genome level presents an opportunity to shed more light on the history of African-Eurasian mixtures, including whether or not selection after admixture is a widespread phenomenon in Africa and how the historical events in Chad are related to events that have occurred elsewhere in Africa and the Near East.

In this work, we present a genetic dataset of 480 Chadian, Near Eastern, and European individuals genotyped at 2.5 million SNPs, in addition to high-coverage

[1]Wellcome Trust Sanger Institute, Wellcome Genome Campus, Hinxton, Cambridge CB10 1SA, UK; [2]Institute for Maternal and Child Health, IRCCS Burlo Garofolo, University of Trieste, 34137 Trieste, Italy; [3]Institute of Molecular and Cell Biology, University of Tartu, Tartu 51010, Estonia; [4]Department of Biochemistry and Molecular Biology, Faculty of Medicine and Health Sciences, Sana'a University, Sana'a 19065, Yemen; [5]Department of Nutrition and Dietetics, Harokopio University Athens, Athens 17671, Greece; [6]National Geographic Society, Washington, DC 20036, USA; [7]Department of Integrative Biology, University of Texas at Austin, Austin, TX 78712, USA; [8]Lebanese American University, Chouran, Beirut 1102 2801, Lebanon; [9]Harvard T.H. Chan School of Public Health, Boston, MA 02115, USA
[10]Present address: Karius, Inc., 1505A Adams Drive, Menlo Park, CA 94025, USA
*Correspondence: mh25@sanger.ac.uk (M.H.), cts@sanger.ac.uk (C.T.-S.)
http://dx.doi.org/10.1016/j.ajhg.2016.10.012.
© 2016 The Author(s). This is an open access article under the CC BY license (http://creativecommons.org/licenses/by/4.0/).

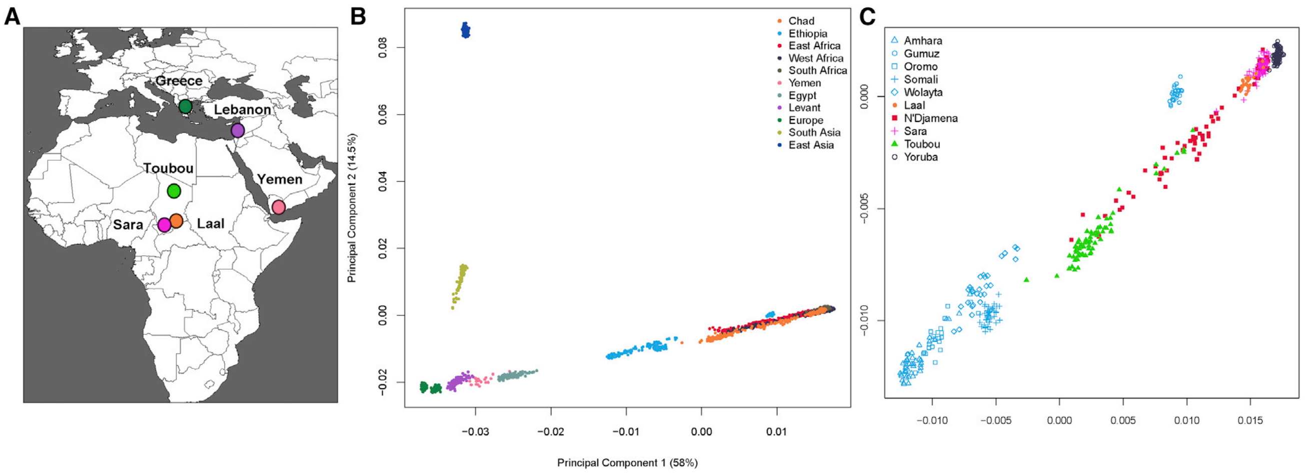

Figure 1. Population Locations and Genetic Structure
(A) The map shows the location of newly genotyped or sequenced populations.
(B) PCA of worldwide populations shows that Near Easterners and East Africans are intermediate to Eurasians and sub-Saharan Africans on PC1. Chad populations are close to sub-Saharan Africans and have some samples drawn toward Ethiopians.
(C) Magnification of the African PCA shows different affinities of the Chad populations to other Africans: the Toubou cluster close to Ethiopians, whereas the Sara and Laal speakers are close to the Yoruba. The mixed samples from N'Djamena, the capital, are intermediate to the Toubou, Sara, and Laal speakers.

whole-genome sequences from 19 of these individuals. From Chad, we studied (1) the Toubou, who are nomads from northern Chad and speak a Nilo-Saharan language; (2) the Sara, who are a sedentary population from southern Chad and also speak a Nilo-Saharan language; (3) the Laal speakers, a population of just ~750 individuals who speak an unclassified language isolate and live in southern Chad; and (4) an urban population from the capital city of N'Djamena. In addition to the Chadians, we included Greek, Lebanese, and Yemen samples whose location and history suggest that they might be informative about early African-Eurasian migrations. We used this dataset to advance our understanding of human genetic diversity in Africa and neighboring regions by focusing on population migration and mixing and how the admixture process has shaped present-day genetic variation.

Subjects and Methods

Samples and Data

Samples were collected from Chad (238), Lebanon (126), Greece (96), and Yemen (20) (Figure 1A); details can be found in Table S1. All samples (except for those from Greece) were genotyped with the Illumina HumanOmni2.5-8 BeadChip, which covers ~2.5 million SNPs. Greek genotype information for the 2.5 million sites was extracted from sequence data (E.Z., unpublished data) and merged with array data from other populations. In addition, 19 samples (Chad [11], Greece [4], and Lebanon [4]) were whole-genome sequenced at >30× depth with Illumina HiSeq X Ten or HiSeq 2500 technology. Genotyping and sequencing were completed at the Wellcome Trust Sanger Institute. Informed consent was obtained from the studied subjects, and the use of the samples in genetic studies was approved by the Human Materials and Data Management Committee at the Wellcome Trust Sanger Institute (approval numbers 09/056 and 14/072) and by the institutional review board (number SMPZ121307-02) of the Lebanese American University.

The genotyping data were merged with data from the African Genome Variation Project,[10] the 1000 Genomes Project,[11] and Pagani et al.,[12] resulting in a combined dataset of ~1.1 million SNPs in 2,453 samples. Analyses including ancient genomes involved merging the panel described above with the Haak et al. dataset,[13] resulting in ~90,000 SNPs in common. Comparative whole-genome sequences were obtained from Pagani et al.[12] and Complete Genomics.[14]

Genotype data were processed with PLINK:[15] the SNP genotype success rate required was set to 99%, whereas SNPs with a minor allele frequency < 0.001 or Hardy-Weinberg p value < 0.000001 were removed. Genotypes from sequence data were called with SAMtools v.1.2[16] and BCFtools v.1.2 with the command "samtools mpileup -q 20 -Q 20 -C 50 | bcftools call -c -V indels." Concordance with array genotypes had a rate of 0.999. Phasing was carried out with SHAPEIT[17] with 1000 Genomes Project phase 3 haplotypes[18] as a reference panel.

Population Structure and Gene Flow

Principal components were computed with EIGENSOFT v.4.2.[19] Effective population size and rates of gene flow were inferred by the multiple sequentially Markovian coalescent (MSMC) approach[20] with four high-coverage phased genomes from each population. We assumed a generation time of 30 years and a mutation rate of 1.25×10^{-8} mutations per nucleotide per generation. Admixture masks to identify African and Eurasian segments within mixed high-coverage genomes were generated with PCAdmix[21] including two ancestral populations based on the 1000 Genomes Project YRI (Yoruba in Ibadan, Nigeria) and CEU (Utah residents with northern and western European ancestry from the CEPH collection) populations. 1 cM windows with a posterior probability of >0.9 for the most likely ancestral state were collected and used for creating African and Eurasian masks.

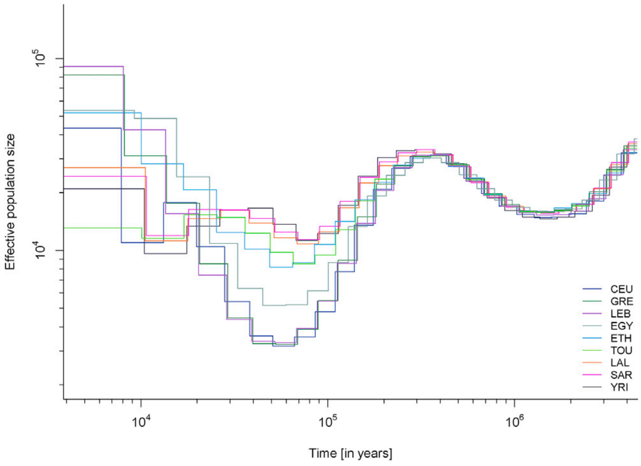

Figure 2. Population-Size Estimates from Whole-Genome Sequences
Population size was inferred by MSMC analysis with four haplotypes from each population. Eurasian populations had a distinctive bottleneck at the time of their exodus from Africa ~60,000 ya. Compared to other Africans, admixed Africans (from a Eurasian gene flow), such as Egyptians, Ethiopians, and the Toubou, also showed a decline in population size during the same period.

Phylogenetic analysis of whole Y chromosome sequences was carried out as described in Bergström et al.[22] Internal node ages were estimated with the rho-statistic[23] and converted to units of years by application of a Y chromosome mutation rate of 0.76×10^{-9} (95% confidence interval [CI] = 0.67×10^{-9} to 0.86×10^{-9}) mutations per site per year.[24] Additionally, Y chromosome haplogroups were defined to the highest resolution possible with 636 SNPs from the array data that overlapped International Society of Genetic Genealogy (ISOGG) markers.

Admixture Analysis

Population-mixture signals and proportions were tested with qp3Pop, qpDstat, and qpF4Ratio from the ADMIXTOOLS package.[25] Admixture proportions were additionally estimated with ADMIXTURE v.1.3.0.[26] ALDER[27] and MALDER[28] were used to date the time of admixture with all pairs of African-Eurasian populations as references. Significant results with a p value < 0.01 were collected and plotted.

Measure of Heterozygosity and Simulations

Heterozygosity on a per-individual basis was estimated with VCFtools v.0.1.13[29] for ~2.17 Gb of the uniquely mappable genome.[11] Heterozygosity was also estimated after correction for recent inbreeding via the removal of long runs of homozygosity (>2 Mb). We investigated the effect of gene flow on the observed heterozygosity by using individual-based forward-time simulations implemented in SimuPOP v.1.1.7.[30]

Selection after Admixture

Evidence for positive selection was tested with the population branch statistic (PBS)[31] with correction for the long-term effective population size.[32] We constructed a tree with the Toubou population branching from the Laal speakers and the Chinese Han as an outgroup. We collected values above the 95th percentile of the PBS distribution and looked for variants previously reported under putative selection in Europeans.

Results

Genetic Structure in Chad Indicates a Complex Admixture History

We performed an initial exploration of our dataset by using principal-component analysis (PCA).[19] The first component (PC1) captured the genetic differentiation between Africans and Eurasians (Figure 1B). Populations such as the Near Easterners and North and East Africans fell between the Europeans and sub-Saharan Africans. The Chadian groups lay near the sub-Saharan Africans: the Sara and Laal speakers clustered tightly with sub-Saharan Africans, such as the Yoruba, whereas the Toubou were somewhat more distant and appeared drawn toward East Africans, such as the Ethiopians. Samples collected from the capital of Chad, N'Djamena, appeared in a central position between the Toubou cluster and the Sara and Laal cluster (Figure 1C). Many individuals from N'Djamena have not reported their ethnicity or have reported a mixed ethnic origin. Therefore, recent mixture could be responsible for their position on the PCA.

We further investigated the genetic variation in Chad by estimating changes in the effective populations size (N_e) over time via the MSMC approach.[20] Eurasians and Africans diverged around 60,000–80,000 ya and subsequently had different patterns of population-size changes: in particular, compared with Africans, the Eurasian population experienced a sharp decrease in size ~60,000 ya.[20] We observed this expected pattern in most populations in our dataset (Figure 2), but a few stood out: (1) Egyptians had a population bottleneck that was much more pronounced than that of other Africans but not as sharp as that of Eurasian populations; and (2) the Toubou and Ethiopians shared a very similar pattern during the bottleneck: they were close to other Africans but had a somewhat sharper decrease in population size (Figure 2). We would not expect such different fluctuations in population sizes at 60,000 ya in populations who shared a common origin during this period. For example, all Eurasians trace their origin to a population who exited Africa ~60,000 ya, and this is reflected in indistinguishable N_e patterns during this period,[20,33] which we also observed in the CEU, Greeks, and Lebanese (Figure 2), as expected. A shared pattern of N_e in ancient times was also observed in the Sara, Laal speakers, and other Africans, such as the Yoruba. We suggest that the deviation from the expected N_e pattern in the Toubou is related to extensive admixture history with Eurasians, like the Eurasian admixture seen in Ethiopians, and we explore this possibility directly with admixture tests below.

Multiple Eurasian Admixtures in Africa after 6,000 ya

We have previously reported massive gene flow ~3,000 ya from Eurasians to Ethiopian populations.[4] Here, we reassess the presence of Eurasian ancestry in Africa by using f_3 statistics[25] in the form of f_3(X; Eurasian, Yoruba), where

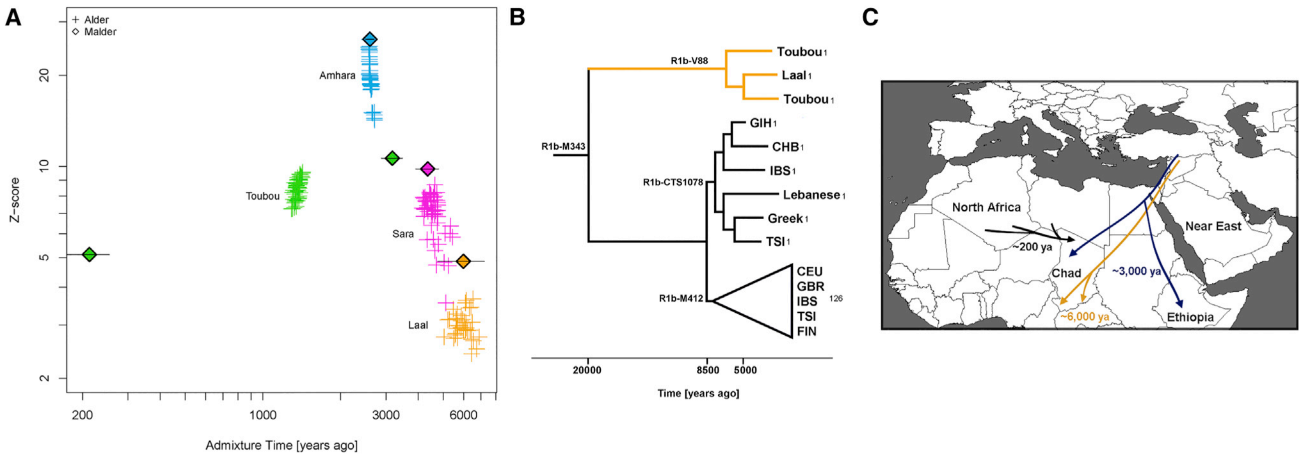

Figure 3. Timing of the Eurasian Admixture in Africa
(A) Crosses represent significant admixture events in the history of the Toubou, Amhara, Sara, and Laal speakers. Time of admixture is estimated from LD by ALDER with all pairs of African-Eurasian populations in our dataset as references. MALDER extends ALDER inference by detecting multiple mixture events, such as in the case of the Toubou population (shown here in green lozenges).
(B) A maximum-likelihood tree shows the males belonging to haplogroup R1b in the 1000 Genomes Project and the R1b males in our dataset. The number of samples is shown on each branch tip. We estimate that the Chadian R1b emerged 5,700–7,300 ya, whereas most European R1b haplogroups emerged 7,300–9,400 ya. The African and Eurasian lineages coalesced 17,900–23,000 ya.
(C) Putative sources and times of admixture of the Eurasian ancestry in Chad and East Africa.

a negative value with a Z score < -4 indicates that X is a mixture of Africans and Eurasians. We found, as expected, that most Ethiopians are a mixture of Africans and Eurasians. An exception is the Gumuz population, where f_3(Gumuz; Eurasian, Yoruba) is always positive. The Gumuz language belongs to the Nilo-Saharan family, which could have isolated the Gumuz from the Afro-Asiatic-speaking Ethiopians. However, we found that the Toubou in Chad, who also speak a Nilo-Saharan language, are a mixture of Africans and Eurasians, making f_3(Toubou; Eurasian, Yoruba) always significantly negative. This suggests that the impact of Eurasian migrations today extends beyond East Africa and the Afro-Asiatic-speaking populations. We did not detect significant (Z score < -4) Eurasian admixture in the Sara (Nilo-Saharan language family) or the Laal speakers (unclassified language) with the use of f_3 statistics (lowest Z score for the Sara was > -2.9; for the Laal speakers, Z scores were all positive). However, this statistic loses sensitivity with small mixture proportions and post-admixture drift,[27] so positive values from the f_3 statistics do not necessarily reflect a complete absence of admixture. We thus further tested for admixture by using ALDER and MALDER, which assess admixture-induced linkage disequilibrium (LD) and can detect small mixture proportions from a substantially diverged reference possibly missed by the f_3 statistic. ALDER detected admixture in the Toubou, Sara, and Laal speakers (Table S2). MALDER, which has the potential to determine whether or not the admixture LD in the population is best represented as the result of one or multiple mixtures, showed that two mixture events had occurred in the Toubou (Figure 3A; Table S3). The first event occurred 2,850–3,500 ya (Z score = 11), a time close to the date of mixture in East Africans 2,500–2,700 ya (Z score = 26). The second mixture event occurred much more recently at 170–260 ya (Z score = 5). In southern Chad, we detected mixture events that were more ancient than those in the north. Mixture occurred 3,900–4,800 ya (Z score = 10) in the Sara and 4,750–7,200 ya (Z score = 5) in the Laal speakers (Figure 3A). These time estimates overlap, and we interpret them as signals from the same admixture event, whose time in the distant past was estimated more reliably in the Laal speakers because they carry more Eurasian ancestry (1.25%–4.5%) than the Sara (0.3%–2%) (see estimates of admixture proportions below), even though the Sara have smaller standard errors because of their larger sample size. In particular, we suggest that the Eurasian mixture event in the Sara and Laal speakers is independent of the mixture event in East Africans and the Toubou for two reasons: (1) admixture LD showed that the events in southern Chad preceded the events in East Africa by 2,000–4,500 years, and (2) we found in Chad a Eurasian Y chromosome lineage (Y haplogroup R1b-V88) that had penetrated all Chadian populations examined but was absent or rare from the Ethiopians examined (Table S4; Figure S1). From whole Y chromosome sequences (Figure S2), we estimate that the Chadian R1b-V88 chromosomes sampled emerged 5,700–7,300 ya (Figure 3B), a time comparable to the Laal speaker admixture dates (4,750–7,200 ya) estimated from genome-wide LD-decay patterns.

The Sources of Eurasian Backflow into Chad and East Africa Are Correlated

Previous studies have suggested that the Eurasian backflow into East Africa came from a population related to early Neolithic farmers.[5] We wanted to know whether the Eurasian ancestry we found in the Toubou, which we

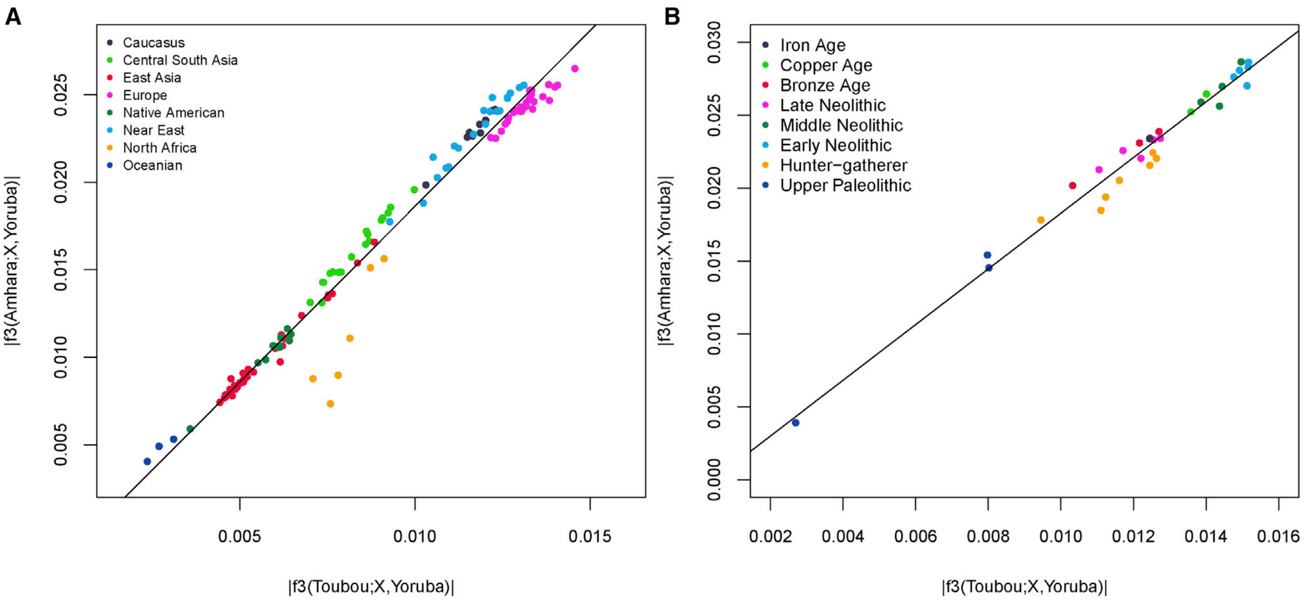

Figure 4. Sources of the Eurasian Ancestry in Chad and Ethiopia
The plot shows significant Eurasian sources for the Toubou and Amhara according to a three-population test (Z score < -4). An increase in the absolute value of the f_3 statistic implies an increase in the genetic affinity of the Eurasian population X to the Toubou and Amhara.
(A) With the exception of North Africans, who showed increased affinity to the Toubou, present-day populations showed correlated affinity to both the Toubou and Amhara. Among modern populations, Sardinians showed the highest genetic affinity to both the Toubou and Amhara.
(B) Ancient Eurasians also showed correlated affinity to both the Toubou and Amhara; the early Neolithic LBK (Linearbandkeramik, or Linear Pottery) population (~5,000 BCE) had the highest affinity.

attribute to a mixture close in time to the date of mixture in East Africans, can be traced to the same source populations that influenced Ethiopia. We performed the tests f_3(Toubou; Yoruba, X) and f_3(Amhara; Yoruba, X), where X is a present-day non-sub-Saharan African population in our dataset and is related to one that contributed ancestry to the Toubou and Amhara (Z score < -4) (Table S5). We then looked at the correlation of the f_3 statistic values between the two tests (Figure 4A). We found that the Eurasian source populations for the Amhara and Toubou were highly correlated ($r = 0.98$; 95% CI = 0.98–0.99; p value $< 2.2 \times 10^{-16}$) and that the most significant result was for present-day Sardinians. Exceptions to this correlation were the North African populations (Tunisians, Mozabite, Algerians, and Saharawi), who appeared to have contributed more ancestry to the Toubou than to the Amhara. We repeated the tests by using published ancient genomes (Table S6) and also found a high correlation of the Eurasian sources for the Amhara and Toubou ($r = 0.98$; 95% CI = 0.97–0.99; p value $< 2.2 \times 10^{-16}$); early Neolithic farmers were the most significant contributors, as reported previously[5] (Figure 4B). When we substituted the Amhara with other Ethiopians (Wolayta and Oromo), we found similar results (data not shown). In a parallel comparison, we checked whether the sources of the African ancestry in different Near Eastern populations were also correlated. We tested f_3(Lebanese; British, X) and f_3(Yemeni; British, X) and found a lower correlation of the f_3 values ($r = 0.62$; 95% CI = 0.32–0.80), suggesting a more complicated history of gene flow from genetically different Africans to different populations in the Near East.

We next quantified the proportion of African-Eurasian mixture in the study populations by using two methods: (1) ADMIXTURE[26] supervised with $K = 2$ and the British and Yoruba as ancestral populations and (2) the f_4 ratio $\alpha = f_4$(British, chimp; X, Yoruba)/f_4(British, chimp; early Neolithic farmer, Yoruba), where X is one of the populations in our dataset (Figure S3). The results from the two tests were highly correlated ($r = 0.998$; 95% CI = 0.996–0.999; p value $< 2.2 \times 10^{-16}$). Eurasian ancestry was estimated at 26%–30% in the Toubou, 0.3%–2% in the Sara, and 1.2%–4.5% in the Laal speakers. Eurasian ancestry in Ethiopians ranged from 11%–12% in the Gumuz to 53%–57% in the Amhara. African ancestry in the Near East ranged from 7%–14% (Yemen) to 0.7%–5% (Lebanese Christians).

Eurasian Gene Flow Shaped the Genomes of Admixed Africans

Our results from the PCA and MSMC analysis showed a deviation of the admixed populations from the patterns observed in unadmixed (or less admixed) populations in the same geographical region. The MSMC analysis, in particular, showed that admixed Africans had patterns indicative of a decline in heterozygosity (increased bottleneck ~60,000 ya), somewhat similarly to Eurasians. We tested whole-genome heterozygosity in these populations and found that it decreased in admixed Africans according

to their Eurasian ancestry (Figure S4A). This decrease was not related to recent inbreeding, given that removing segments with long runs of homozygosity did not change the overall pattern. Our simulations suggest that decay in heterozygosity is expected after gene flow from a population with diversity comparable to that of Eurasians (Figures S4B and S4C). We further investigated heterozygosity in admixed Africans by assessing heterozygosity of the different ancestral segments in the Toubou genome. We found that admixed African-Eurasian segments had more heterozygosity (1.23 hets/kb) than segments of the genome where African-African haplotypes were present (1.19 hets/kb) (Figure S5). However, the Toubou genome segments with complete Eurasian ancestry (Eurasian-Eurasian) had considerably lower heterozygosity (~0.96 hets/kb; Figure S5), leading to the genome-wide pattern of decay in heterozygosity observed in Africans with Eurasian ancestry (Figure S4).

We wanted to understand the consequence of admixture on the models that use the density of heterozygous sites to infer the demographic history of populations. We first tested whether the coalescent history estimated by MSMC was affected by a small proportion of mixture, such as the African mixture found in Greeks and Lebanese (ranging from 0% to 5%). We tested the Greek, Lebanese, CEU, and CHB (Han Chinese in Beijing, China) split times from the Yoruba and found that all populations split from the Yoruba ~70,000–80,000 ya, implying that the low proportions of African admixture in the Greeks and Lebanese did not detectably affect the estimates of relative cross-coalescence rate (Figure S6A). We next tested the Toubou, who have ~30% Eurasian ancestry. The Toubou appeared to split from Eurasians ~30,000–40,000 ya, a time more recent than expected considering the African-Eurasian split 60,000–80,000 ya[20] (Figure S6B). We tested other Africans in our dataset and found that the Sara, Laal speakers, and Yoruba split from Eurasians, as expected, ~70,000–80,000 ya (Figure S6B). We then tested directly whether the Eurasian ancestry affected the relative cross-coalescence rate between the Toubou and Eurasians by masking some of the Eurasian ancestry in the Toubou. We used PCAdmix[21] to estimate the ancestry along each chromosome and then used the identified Eurasian segments as a negative mask in our analysis. The split times between the Toubou and Lebanese, for example, increased by ~15,000 years (Figure S6B), shifting the split date toward the expected African-Eurasian split time.

We found that, in addition to influencing the relative cross-coalescence rate, admixture can also inflate putative signals of positive selection. For example, using the PBS[31] to detect recent positive selection that occurred in the Toubou after their divergence from the Yoruba, we found signals of selection on *MCM6* (MIM: 601806) rs4988235, a variant associated with the lactase-persistence phenotype. This SNP was previously found to be under strong positive selection in Europeans, where it was probably advantageous to individuals living in pastoralist societies.[34] The frequency of this variant in the Toubou is 2%, and it is absent from the sub-Saharan African and other Chadic samples (the Sara and Laal speakers) examined here. Although this SNP appears to be a candidate for selection, we suggest that it has probably drifted neutrally in the Toubou after the Eurasian gene flow: the Toubou have ~30% Eurasian ancestry from a population similar to the Greeks, who have 13% derived alleles at rs4988235, suggesting an expectation of ~3.9% of the derived allele simply from admixture. We similarly found in the Toubou signals at *HERC2* (MIM: 605837) rs1129038 a major contributor to blue eye color in Europeans[35] (Toubou derived allele frequency [DAF] = 0.014; Greek DAF = 0.33; Yoruba, Sara, and Laal DAF = 0), as well as a signal at *SLC24A5* (MIM: 609802) rs1834640, a major contributor to pigmentation[36] (Toubou DAF = 0.19; Greek DAF = 0.99; Yoruba, Sara, and Laal DAF = 0–0.04).

In addition to introducing to African populations genes that were positively selected in Europe, the recent African-Eurasian admixture carried Neanderthal alleles to Central and East Africa. Neanderthals are closer to the Amhara than to the Yoruba: *D*(Neanderthal, chimp, Amhara, Yoruba) = 0.0094. Neanderthals are also closer to the Toubou than to the Yoruba: *D*(Neanderthal, chimp, Toubou, Yoruba) = 0.0041. On the other hand, we found that Neanderthals are closer to Europeans than to Near Easterners: *D*(Neanderthal, chimp; French, Yemen) = 0.0056 and *D*(Neanderthal, chimp; French, Palestinian) = 0.0040.

We estimated the archaic ancestry proportions by using the ratio

$$\alpha = \frac{f4(\mathrm{Altai, chimp}; X, \mathrm{Yoruba})}{f4(\mathrm{Altai, chimp; Vindija, Yoruba})}$$

and found that Neanderthal ancestry was ~0.5% in the Toubou and~1% in the Amhara. We then computed the correlation between the Neanderthal ancestry proportions and the Eurasian and African ancestry proportions we identified. Neanderthal ancestry in admixed Africans and Near Easterners was highly correlated with their Eurasian ancestry ($r = 0.98$; p value $< 2.2 \times 10^{-16}$) and inversely correlated with their African ancestry (Figure 5).

Discussion

We have generated an extensive set of genotyping and high-coverage whole-genome sequencing data to study the genetic history of Chad and neighboring populations. We found substantial genetic differences between the ethnic groups inhabiting Chad today and suggest that multiple ancient Eurasian migrations played a major role in shaping the genetic diversity of the region (Figure 3C). Here, we discuss these migrations and how the mixed ancestry can confound proper interpretation of the evolutionary processes that occurred in their history and therefore needs to be thoroughly accounted for in the study of African genetic diversity.

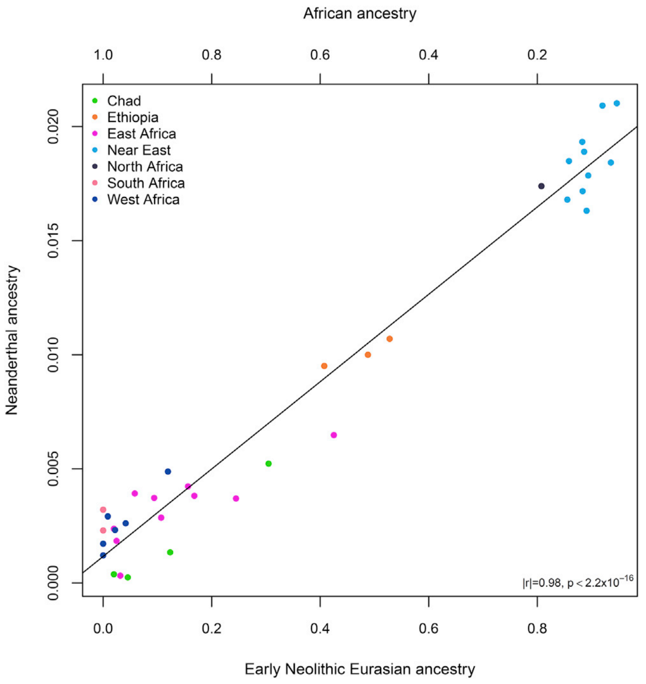

Figure 5. Neanderthal Ancestry Correlation with the African-Eurasian Admixture

Neanderthal ancestry is not expected in Africa, yet today many Africans carry Neanderthal-derived alleles. The plot shows that the Neanderthal ancestry proportion in Africans is correlated with gene flow from Eurasians. For example, knowing that today Eurasians carry ~2% of Neanderthal ancestry, we observed that East Africans (Ethiopians) had ~1% Neanderthal ancestry and ~50% Eurasian ancestry. Correspondingly, Near Easterners showed a decline in Neanderthal ancestry proportional to their levels of African ancestry.

We detected the earliest Eurasian migrations to Africa in the Laal-speaking people, an isolated language group of fewer than 800 speakers who inhabit southern Chad. We estimate that mixture occurred 4,750–7,200 ya, thus after the Neolithic transition in the Near East, a period characterized by exponential growth in human population size. Environmental changes during this period (which possibly triggered the Neolithic transition) also facilitated human migrations. The African Humid Period, for example, was a humid phase across North Africa that peaked 6,000–9,000 ya[37] and biogeographically connected Africa to Eurasia, facilitating human movement across these regions.[38] In Chad, we found a Y chromosome lineage (R1b-V88) that we estimate emerged during the same period 5,700–7,300 ya (Figure 3B). The closest related Y chromosome groups today are widespread in Eurasia and have been previously associated with human expansions to Europe.[39,40] We estimate that the Eurasian R1b lineages initially diverged 7,300–9,400 ya, at the time of the Neolithic expansions. However, we found that the African and Eurasian R1b lineages diverged 17,900–23,000 ya, suggesting that genetic structure was already established between the groups who expanded to Europe and Africa. R1b-V88 was previously found in Central and West Africa and was associated with a mid-Holocene migration of Afro-asiatic speakers through the central Sahara into the Lake Chad Basin.[8] In the populations we examined, we found R1b in the Toubou and Sara, who speak Nilo-Saharan languages, and also in the Laal people, who speak an unclassified language. This suggests that R1b penetrated Africa independently of the Afro-asiatic language spread or passed to other groups through admixture.

In addition to the early Eurasian migration to Africa ~6,000 ya, a second migration ~3,000 ya affected the Toubou population in northern Chad but had no detectable genetic impact on other Chadian populations. This migration appears to be associated with the previously reported Eurasian backflow into East Africa, given that the source populations and dates of mixture are similar. Occurring at the start of the Iron Age, these migrations could have been facilitated by advances in warfare and transportation technology in the Near East. It is uncertain why the impact of this migration in Chad affected only the Toubou. The African ancestral component in the Toubou is best represented by the Laal-speaking population, suggesting that the African-Eurasian mixture probably occurred in Chad. However, ethnolinguistic barriers could have already been established at this time between the Chad groups, preventing a widespread dissemination of the Eurasian ancestry. The Toubou, despite their Islamic faith, do not show the genetic admixture detected in many Near Eastern and North African populations around 1,100 ya,[41] suggesting conversion without population mixing at this time. They did, however, receive additional Eurasian ancestry in the past 200 years from a source represented by North African populations such as Tunisians, Mozabite, Algerians, and Sahrawi (Figure 3C). This recent interaction could have been promoted by the nomadic lifestyle of the present-day Toubou and a shared Muslim religion with North Africans. Unsurprisingly, we also detected a likely mixing of Chad populations in the sample from the capital, which could be even more recent.

Eurasian backflow into Africa thus appears to have been a recurrent event in the history of many Africans, given its considerable impact on their genomes. Although population mixture in general is a process that increases genetic diversity, we observed a decrease in heterozygosity in the admixed Africans. Our simulations showed that these results are expected after mixture at these proportions with the Eurasians who suffered a significant bottleneck at the time of their exodus from Africa ~60,000 ya. Consequently, we found that mixture can complicate interpretation of the coalescent history inferred from models that use the density of heterozygous sites in their implementations. In addition, we detected in admixed Africans an inflation of positive-selection signals on alleles associated with adult lactose tolerance and pigmentation in Europeans, but we suggest that these alleles have drifted neutrally in Africans after admixture. Furthermore, we detected Neanderthal ancestry in admixed Africans and found it to be proportional to their Eurasian ancestry.

Similarly, in admixed Near Easterners, we found a decrease in Neanderthal ancestry proportional to the gene flow they have received from Africans. Although a higher genetic affinity of Neanderthals to Europeans than to Near Easterners was previously interpreted as additional Neanderthal admixture in the history of Europeans,[42] we propose that a more parsimonious explanation for these observations is that African-Eurasian mixtures both introduced Neanderthal ancestry to Africa and "diluted" the Neanderthal ancestry in the Near East.

It is important to note that in this work we inevitably invoke Occam's razor to support the simplest model consistent with our data; the history of the populations studied here, including the time and sources of the Eurasian admixture in Africa, could be more complex. aDNA from Chad and neighboring regions remains a challenge given the poor DNA preservation in hot climates, but future successful efforts in aDNA research could provide additional insights and reveal additional complexities not considered by the modern-DNA-based models favored here.[43]

Our study has shown that human genetic diversity in Africa is still incompletely understood and that ancient admixture adds to its complexity. This work highlights the importance of exploring underrepresented populations, such as those from Chad, in genetic studies to improve our understanding of the demographic processes that shaped genetic variation in Africa and globally.

Accession Numbers

Whole-genome sequencing and SNP genotyping data are available through the European Genome-phenome Archive (EGA) under accession numbers EGA: EGAD00001002742 (sequences from Chad and Lebanon), EGAD00001001440 (sequences from Greece), and EGAS00001001231 (SNP data from Chad, Lebanon, and Yemen).

Supplemental Data

Supplemental Data include six figures and six tables and can be found with this article online at http://dx.doi.org/10.1016/j.ajhg.2016.10.012.

Acknowledgments

We thank all sample donors for making this work possible and the Wellcome Trust Sanger Institute pipelines for generating genotype and sequence data. We also thank Andrea Massaia for comments on analyzing Y chromosome haplogroups from array data and David Soria-Hernanz for comments on the ethnic groups and languages in Chad. M.H., M.M., A.B., J.P.-M., E.Z., Y.X., and C.T.-S. were supported by the Wellcome Trust (098051). P.H. was supported by Estonian Research Council grant PUT1036. J.B.-S. is a full-time employee of Karius, Inc., and R.S.W. is founder and CEO of Insitome.

Received: August 15, 2016
Accepted: October 24, 2016
Published: November 23, 2016

Web Resources

European Genome-phenome Archive (EGA), https://www.ebi.ac.uk/ega/home

ISOGG Y-DNA Haplotype Tree v.11.201, http://www.isogg.org/tree/

References

1. Central Intelligence Agency. (2014). The World Factbook 2014. https://www.cia.gov/library/publications/the-world-factbook/index.html.
2. Kröpelin, S., Verschuren, D., Lézine, A.M., Eggermont, H., Cocquyt, C., Francus, P., Cazet, J.P., Fagot, M., Rumes, B., Russell, J.M., et al. (2008). Climate-driven ecosystem succession in the Sahara: the past 6000 years. Science *320*, 765–768.
3. Löhr, D. (2009). Lake Chad and the migratory routes to Borno: A linguistic trail. In Migrations and Spatial Mobility in the Lake Chad Basin, XIIIth Mega-Chad Conference, H. Tourneaux, ed. (Editions de l'IRD), pp. 665–681.
4. Pagani, L., Kivisild, T., Tarekegn, A., Ekong, R., Plaster, C., Gallego Romero, I., Ayub, Q., Mehdi, S.Q., Thomas, M.G., Luiselli, D., et al. (2012). Ethiopian genetic diversity reveals linguistic stratification and complex influences on the Ethiopian gene pool. Am. J. Hum. Genet. *91*, 83–96.
5. Gallego Llorente, M., Jones, E.R., Eriksson, A., Siska, V., Arthur, K.W., Arthur, J.W., Curtis, M.C., Stock, J.T., Coltorti, M., Pieruccini, P., et al. (2015). Ancient Ethiopian genome reveals extensive Eurasian admixture in Eastern Africa. Science *350*, 820–822.
6. Cerný, V., Fernandes, V., Costa, M.D., Hájek, M., Mulligan, C.J., and Pereira, L. (2009). Migration of Chadic speaking pastoralists within Africa based on population structure of Chad Basin and phylogeography of mitochondrial L3f haplogroup. BMC Evol. Biol. *9*, 63.
7. Cerezo, M., Černý, V., Carracedo, Á., and Salas, A. (2011). New insights into the Lake Chad Basin population structure revealed by high-throughput genotyping of mitochondrial DNA coding SNPs. PLoS ONE *6*, e18682.
8. Cruciani, F., Trombetta, B., Sellitto, D., Massaia, A., Destro-Bisol, G., Watson, E., Beraud Colomb, E., Dugoujon, J.M., Moral, P., and Scozzari, R. (2010). Human Y chromosome haplogroup R-V88: a paternal genetic record of early mid Holocene trans-Saharan connections and the spread of Chadic languages. Eur. J. Hum. Genet. *18*, 800–807.
9. Triska, P., Soares, P., Patin, E., Fernandes, V., Cerny, V., and Pereira, L. (2015). Extensive Admixture and Selective Pressure Across the Sahel Belt. Genome Biol. Evol. *7*, 3484–3495.
10. Gurdasani, D., Carstensen, T., Tekola-Ayele, F., Pagani, L., Tachmazidou, I., Hatzikotoulas, K., Karthikeyan, S., Iles, L., Pollard, M.O., Choudhury, A., et al. (2015). The African Genome Variation Project shapes medical genetics in Africa. Nature *517*, 327–332.
11. Abecasis, G.R., Altshuler, D., Auton, A., Brooks, L.D., Durbin, R.M., Gibbs, R.A., Hurles, M.E., McVean, G.A.; and 1000 Genomes Project Consortium (2010). A map of human genome variation from population-scale sequencing. Nature *467*, 1061–1073.
12. Pagani, L., Schiffels, S., Gurdasani, D., Danecek, P., Scally, A., Chen, Y., Xue, Y., Haber, M., Ekong, R., Oljira, T., et al. (2015). Tracing the route of modern humans out of Africa by using 225 human genome sequences from Ethiopians and Egyptians. Am. J. Hum. Genet. *96*, 986–991.

13. Haak, W., Lazaridis, I., Patterson, N., Rohland, N., Mallick, S., Llamas, B., Brandt, G., Nordenfelt, S., Harney, E., Stewardson, K., et al. (2015). Massive migration from the steppe was a source for Indo-European languages in Europe. Nature *522*, 207–211.
14. Drmanac, R., Sparks, A.B., Callow, M.J., Halpern, A.L., Burns, N.L., Kermani, B.G., Carnevali, P., Nazarenko, I., Nilsen, G.B., Yeung, G., et al. (2010). Human genome sequencing using unchained base reads on self-assembling DNA nanoarrays. Science *327*, 78–81.
15. Purcell, S., Neale, B., Todd-Brown, K., Thomas, L., Ferreira, M.A., Bender, D., Maller, J., Sklar, P., de Bakker, P.I., Daly, M.J., and Sham, P.C. (2007). PLINK: a tool set for whole-genome association and population-based linkage analyses. Am. J. Hum. Genet. *81*, 559–575.
16. Li, H., Handsaker, B., Wysoker, A., Fennell, T., Ruan, J., Homer, N., Marth, G., Abecasis, G., Durbin, R.; and 1000 Genome Project Data Processing Subgroup (2009). The Sequence Alignment/Map format and SAMtools. Bioinformatics *25*, 2078–2079.
17. Delaneau, O., Zagury, J.F., and Marchini, J. (2013). Improved whole-chromosome phasing for disease and population genetic studies. Nat. Methods *10*, 5–6.
18. Auton, A., Brooks, L.D., Durbin, R.M., Garrison, E.P., Kang, H.M., Korbel, J.O., Marchini, J.L., McCarthy, S., McVean, G.A., Abecasis, G.R.; and 1000 Genomes Project Consortium (2015). A global reference for human genetic variation. Nature *526*, 68–74.
19. Patterson, N., Price, A.L., and Reich, D. (2006). Population structure and eigenanalysis. PLoS Genet. *2*, e190.
20. Schiffels, S., and Durbin, R. (2014). Inferring human population size and separation history from multiple genome sequences. Nat. Genet. *46*, 919–925.
21. Brisbin, A., Bryc, K., Byrnes, J., Zakharia, F., Omberg, L., Degenhardt, J., Reynolds, A., Ostrer, H., Mezey, J.G., and Bustamante, C.D. (2012). PCAdmix: principal components-based assignment of ancestry along each chromosome in individuals with admixed ancestry from two or more populations. Hum. Biol. *84*, 343–364.
22. Bergström, A., Nagle, N., Chen, Y., McCarthy, S., Pollard, M.O., Ayub, Q., Wilcox, S., Wilcox, L., van Oorschot, R.A., McAllister, P., et al. (2016). Deep roots for Aboriginal Australian Y chromosomes. Curr. Biol. *26*, 809–813.
23. Forster, P., Harding, R., Torroni, A., and Bandelt, H.J. (1996). Origin and evolution of Native American mtDNA variation: a reappraisal. Am. J. Hum. Genet. *59*, 935–945.
24. Fu, Q., Li, H., Moorjani, P., Jay, F., Slepchenko, S.M., Bondarev, A.A., Johnson, P.L., Aximu-Petri, A., Prüfer, K., de Filippo, C., et al. (2014). Genome sequence of a 45,000-year-old modern human from western Siberia. Nature *514*, 445–449.
25. Patterson, N., Moorjani, P., Luo, Y., Mallick, S., Rohland, N., Zhan, Y., Genschoreck, T., Webster, T., and Reich, D. (2012). Ancient admixture in human history. Genetics *192*, 1065–1093.
26. Alexander, D.H., Novembre, J., and Lange, K. (2009). Fast model-based estimation of ancestry in unrelated individuals. Genome Res. *19*, 1655–1664.
27. Loh, P.R., Lipson, M., Patterson, N., Moorjani, P., Pickrell, J.K., Reich, D., and Berger, B. (2013). Inferring admixture histories of human populations using linkage disequilibrium. Genetics *193*, 1233–1254.
28. Pickrell, J.K., Patterson, N., Loh, P.R., Lipson, M., Berger, B., Stoneking, M., Pakendorf, B., and Reich, D. (2014). Ancient west Eurasian ancestry in southern and eastern Africa. Proc. Natl. Acad. Sci. USA *111*, 2632–2637.
29. Danecek, P., Auton, A., Abecasis, G., Albers, C.A., Banks, E., DePristo, M.A., Handsaker, R.E., Lunter, G., Marth, G.T., Sherry, S.T., et al.; 1000 Genomes Project Analysis Group (2011). The variant call format and VCFtools. Bioinformatics *27*, 2156–2158.
30. Peng, B., and Kimmel, M. (2005). simuPOP: a forward-time population genetics simulation environment. Bioinformatics *21*, 3686–3687.
31. Yi, X., Liang, Y., Huerta-Sanchez, E., Jin, X., Cuo, Z.X., Pool, J.E., Xu, X., Jiang, H., Vinckenbosch, N., Korneliussen, T.S., et al. (2010). Sequencing of 50 human exomes reveals adaptation to high altitude. Science *329*, 75–78.
32. Ayub, Q., Mezzavilla, M., Pagani, L., Haber, M., Mohyuddin, A., Khaliq, S., Mehdi, S.Q., and Tyler-Smith, C. (2015). The Kalash genetic isolate: ancient divergence, drift, and selection. Am. J. Hum. Genet. *96*, 775–783.
33. Li, H., and Durbin, R. (2011). Inference of human population history from individual whole-genome sequences. Nature *475*, 493–496.
34. Enattah, N.S., Sahi, T., Savilahti, E., Terwilliger, J.D., Peltonen, L., and Järvelä, I. (2002). Identification of a variant associated with adult-type hypolactasia. Nat. Genet. *30*, 233–237.
35. Sturm, R.A., Duffy, D.L., Zhao, Z.Z., Leite, F.P., Stark, M.S., Hayward, N.K., Martin, N.G., and Montgomery, G.W. (2008). A single SNP in an evolutionary conserved region within intron 86 of the *HERC2* gene determines human blue-brown eye color. Am. J. Hum. Genet. *82*, 424–431.
36. Stokowski, R.P., Pant, P.V., Dadd, T., Fereday, A., Hinds, D.A., Jarman, C., Filsell, W., Ginger, R.S., Green, M.R., van der Ouderaa, F.J., and Cox, D.R. (2007). A genomewide association study of skin pigmentation in a South Asian population. Am. J. Hum. Genet. *81*, 1119–1132.
37. deMenocal, P.B., and Tierney, J.E. (2012). Green Sahara: African Humid Periods paced by Earth's orbital changes. Nature Education Knowledge *3*, 12.
38. Larrasoaña, J.C., Roberts, A.P., and Rohling, E.J. (2013). Dynamics of green Sahara periods and their role in hominin evolution. PLoS ONE *8*, e76514.
39. Balaresque, P., Bowden, G.R., Adams, S.M., Leung, H.Y., King, T.E., Rosser, Z.H., Goodwin, J., Moisan, J.P., Richard, C., Millward, A., et al. (2010). A predominantly neolithic origin for European paternal lineages. PLoS Biol. *8*, e1000285.
40. Batini, C., Hallast, P., Zadik, D., Delser, P.M., Benazzo, A., Ghirotto, S., Arroyo-Pardo, E., Cavalleri, G.L., de Knijff, P., Dupuy, B.M., et al. (2015). Large-scale recent expansion of European patrilineages shown by population resequencing. Nat. Commun. *6*, 7152.
41. Haber, M., Gauguier, D., Youhanna, S., Patterson, N., Moorjani, P., Botigué, L.R., Platt, D.E., Matisoo-Smith, E., Soria-Hernanz, D.F., Wells, R.S., et al. (2013). Genome-wide diversity in the levant reveals recent structuring by culture. PLoS Genet. *9*, e1003316.
42. Rodriguez-Flores, J.L., Fakhro, K., Agosto-Perez, F., Ramstetter, M.D., Arbiza, L., Vincent, T.L., Robay, A., Malek, J.A., Suhre, K., Chouchane, L., et al. (2016). Indigenous Arabs are descendants of the earliest split from ancient Eurasian populations. Genome Res. *26*, 151–162.
43. Haber, M., Mezzavilla, M., Xue, Y., and Tyler-Smith, C. (2016). Ancient DNA and the rewriting of human history: be sparing with Occam's razor. Genome Biol. *17*, 1.

The American Journal of Human Genetics, Volume *99*

Supplemental Data

Chad Genetic Diversity Reveals an African History Marked by Multiple Holocene Eurasian Migrations

Marc Haber, Massimo Mezzavilla, Anders Bergström, Javier Prado-Martinez, Pille Hallast, Riyadh Saif-Ali, Molham Al-Habori, George Dedoussis, Eleftheria Zeggini, Jason Blue-Smith, R. Spencer Wells, Yali Xue, Pierre A. Zalloua, and Chris Tyler-Smith

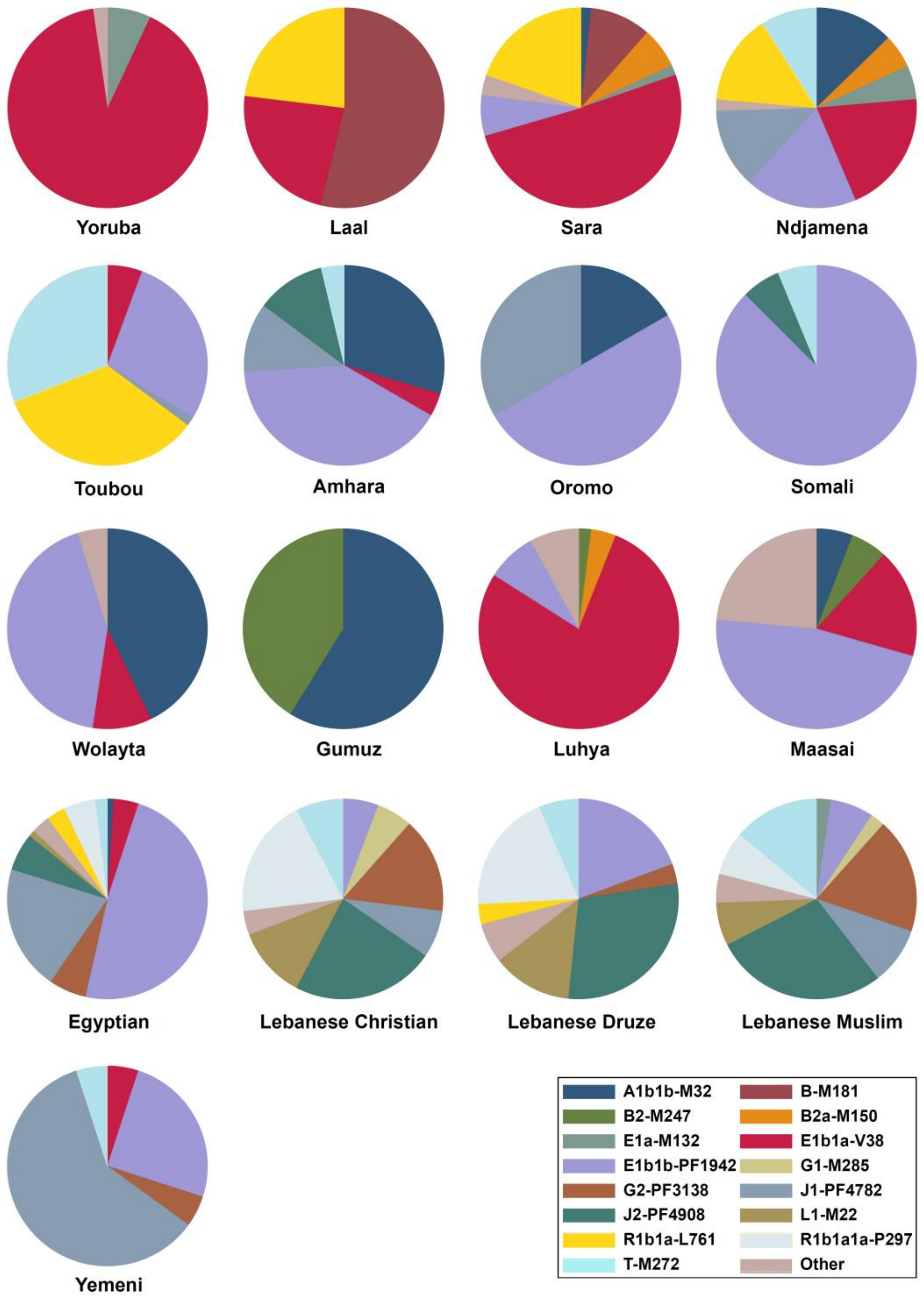

Figure S1. Y-chromosome haplogroup distribution. We find in Chad a high frequency of Y-haplogroup R1b in all ethnic groups examined. On the other hand, R1b is absent or rare in East African populations. We also find the Eurasian haplogroup T in Toubou and Ethiopians with Toubou having a high frequency (31%) of their studied males belonging to this haplogroup. Interestingly, the only instances of this haplogroup in examined ancient populations are in the *Linearbandkeramik* (LBK) population which we found to be the most significant reference for the Eurasian ancestry in Toubou and the Ethiopians. For more detailed haplogroup definitions see Table S2. Category "Other" contains haplogroups B2b, E, E2, I2, Q, R1a and R2a.

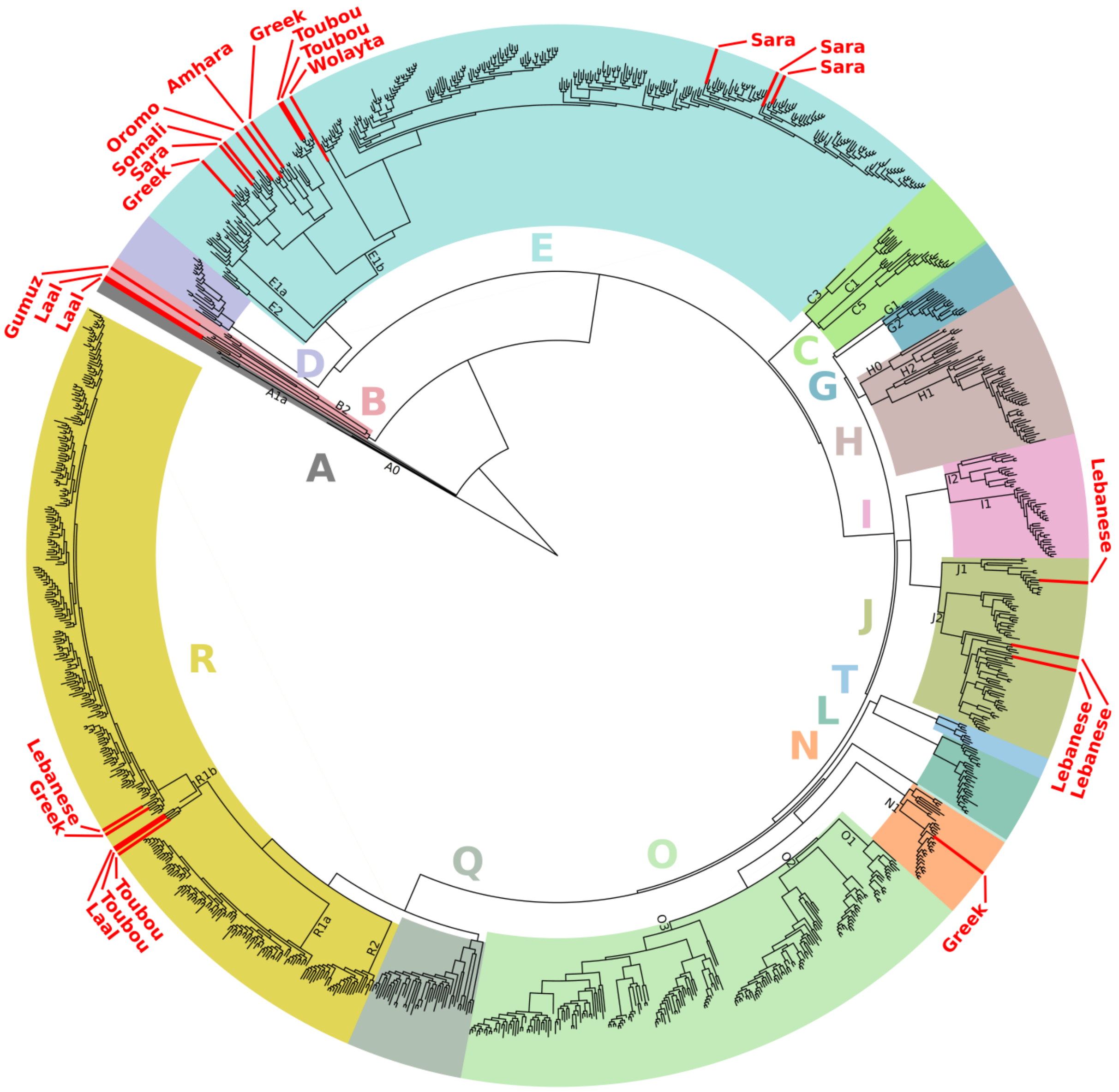

Figure S2. Y-chromosome sequences in a global phylogenetic context. A maximum likelihood phylogenetic tree of the sequenced Y chromosomes in our dataset and those of all the 1244 males from the 1000 Genomes Project. Coloured ranges correspond to high level haplogroups. Some finer-scale haplogroup information is indicated by labels on the tree. The samples sequenced in this study are highlighted in red.

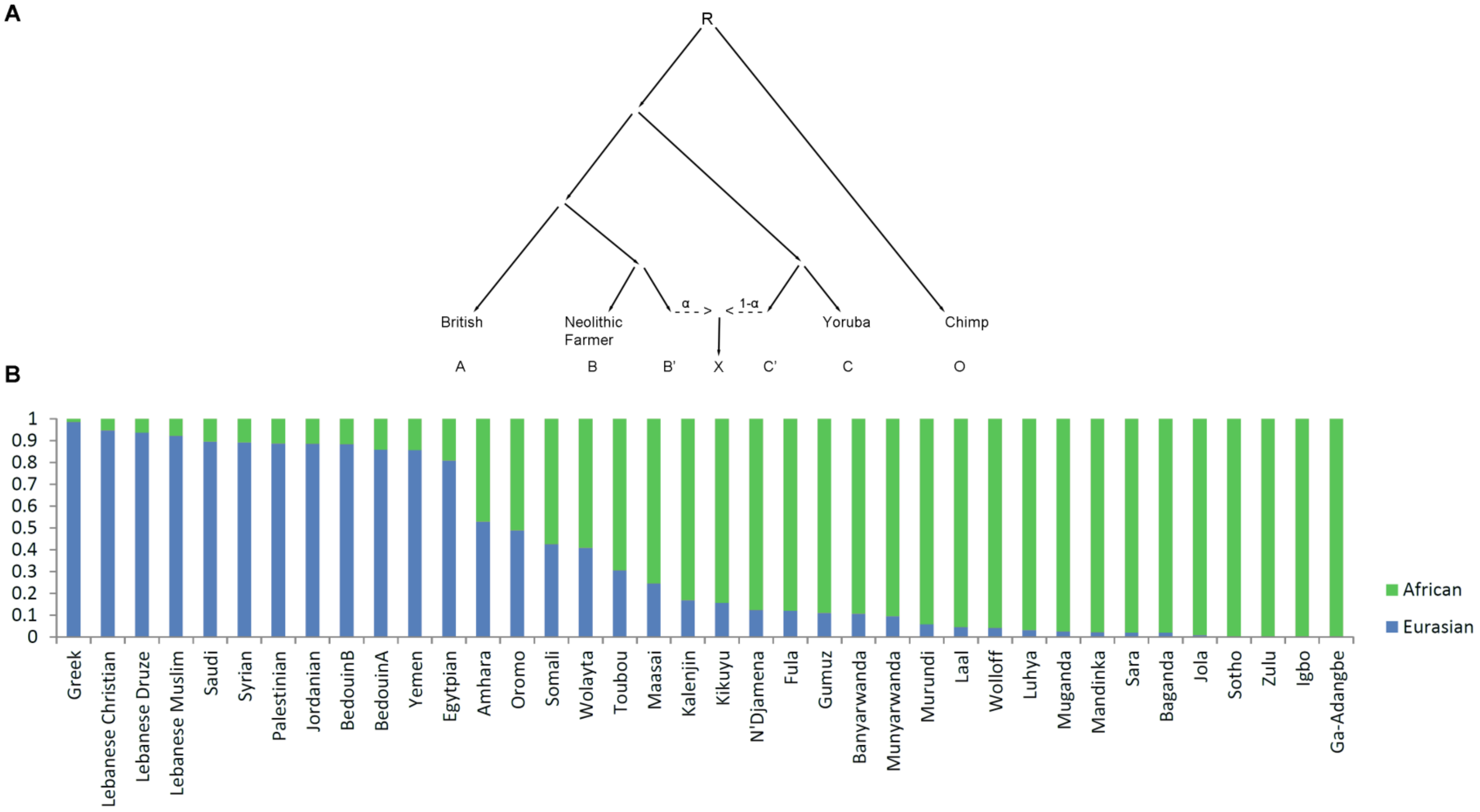

Figure S3. African-Eurasian mixture proportions. We quantify the proportion of admixture using the f_4 ratio, with populations related to European Neolithic farmers and Yoruba as source populations. Eurasian ancestry in Chad ranges from 0.3-2% in Sara to 26-30% in Toubou. A) Population phylogeny used to estimate the African-Eurasian admixture proportions. B) Estimated African-Eurasian ancestry proportions in present-day populations.

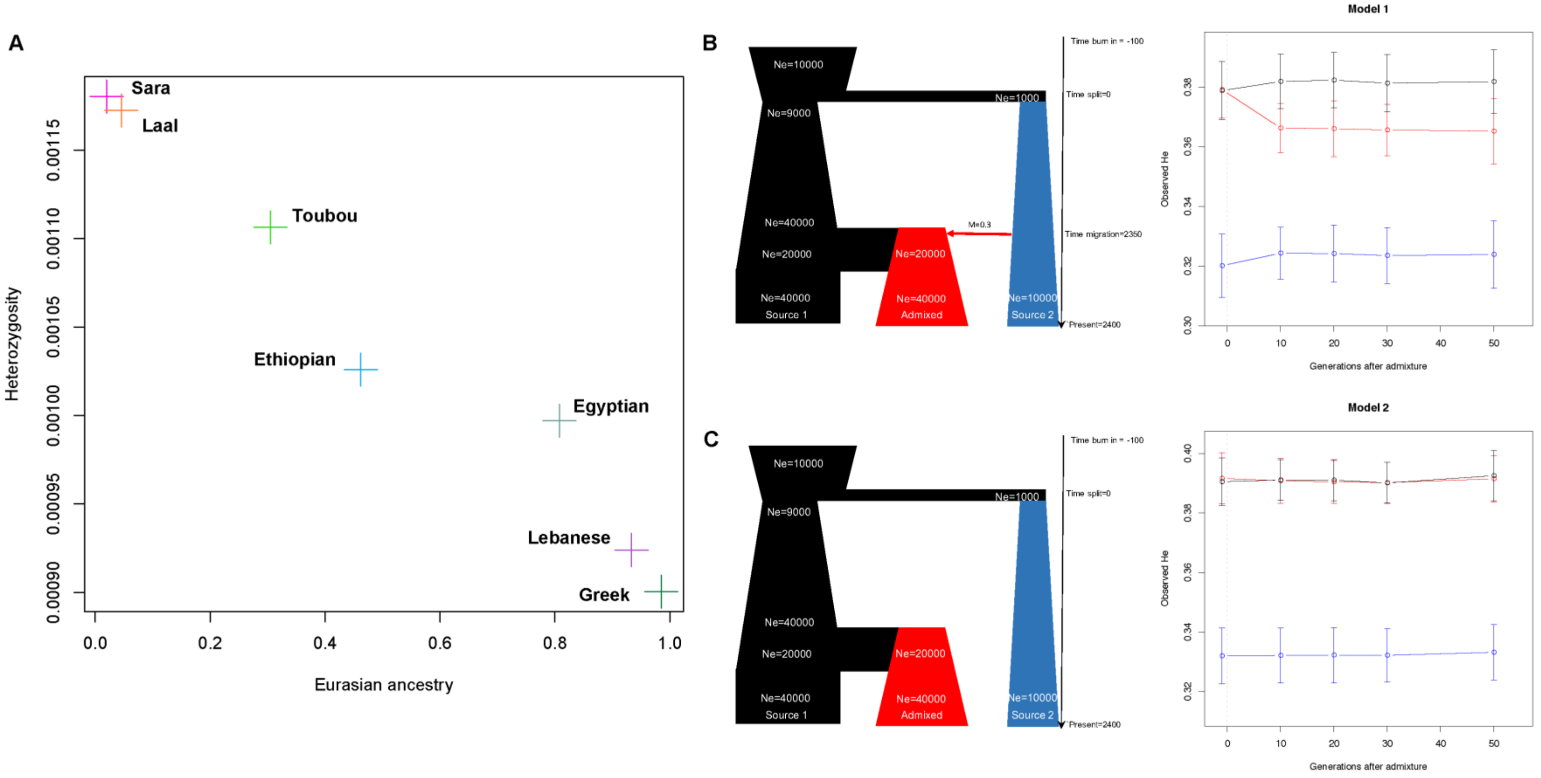

Figure S4. Eurasian admixture consequence for heterozygosity. A) Heterozygosity decline in our whole-genome sequence data is correlated with the proportion of Eurasian ancestry in the population. B) Simulations show that a decrease in heterozygosity is expected after 30% mixture with a population who had a bottleneck in their history, in a model incorporating the decrease in population size in the Eurasians during their exit from Africa (Model 1). C) Structure in the African population without admixture cannot explain the decline in heterozygosity observed in the populations examined (Model 2). Observed He in B and C is mean He estimated across 1000 simulated loci.

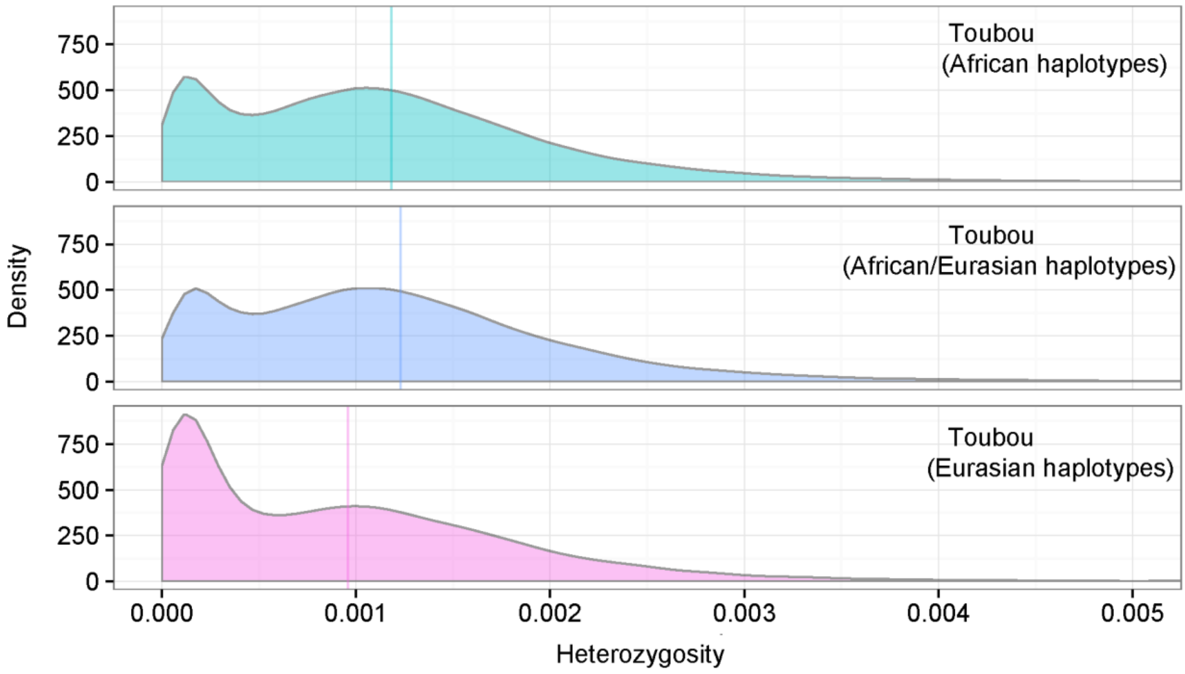

Figure S5. Distribution of heterozygosity in 100kbp windows in whole-genome sequence data from four Toubou individuals. Plots show segments of the Toubou genomes corresponding to different ancestries putatively assigned with PCAdmix. The mean of the distributions is plotted in a vertical line. Admixed African/Eurasian ancestral segments have increased heterozygosity compared with unadmixed African/African segments. However, the Toubou genome segments with complete Eurasian ancestry (Eurasian/Eurasian) have considerable decrease in heterozygosity (~0.96 hets/kbp), leading to the genome-wide decay in heterozygosity pattern observed in Africans with Eurasian ancestry.

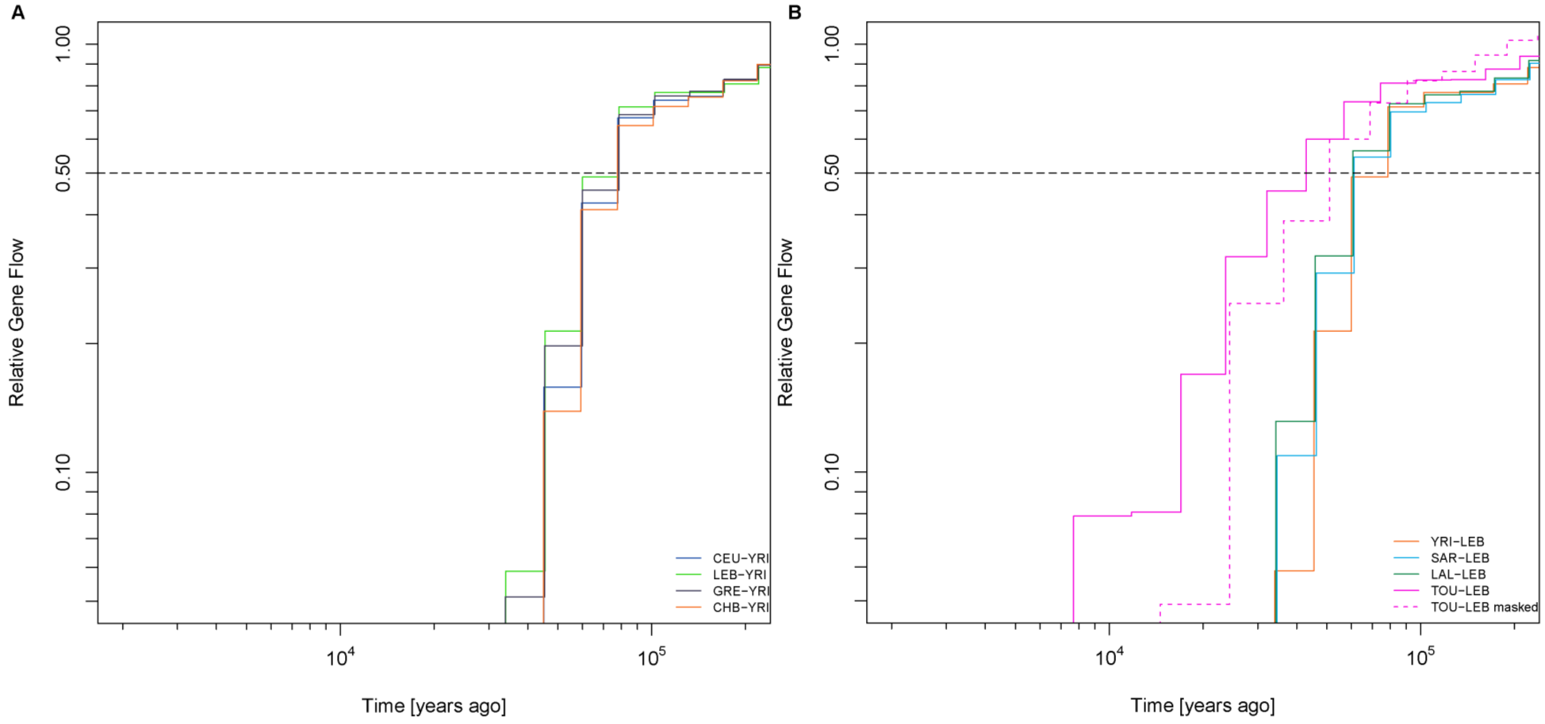

Figure S6. Effect of admixture on the relative cross coalescence rates. Plots show relative gene flow between pairs of populations where a decrease below 50% is assumed to be the time of divergence A) a small proportion of African ancestry <5% in Greek and Lebanese do not bias the inferences from MSMC on their split time from Yoruba, which remains identical to the split time of CEU and CHB from Yoruba. B) A significant proportion of Eurasian ancestry in Toubou (~30%) appears to change their split times from Eurasians towards more recent times compared with other Africans such as Yoruba, Sara, and Laal. Masking part of the Eurasian ancestry in Toubou increases their time of divergence with Eurasians closer to the time of the African-Eurasian split.

Table S1. New populations and data analysed in this paper.

Population	Language family	Location	Country	Region	2.5M array[a]	WGS[a]
Laal	Unclassified	Moyen-Chari	Chad	Africa	26	3
Sara	Nilo-Saharan	Moyen-Chari	Chad	Africa	62	4
Toubou	Nilo-Saharan	Borkou-Ennedi-Tibesti	Chad	Africa	83	4
N'Djamena	Mix	N'Djamena	Chad	Africa	67	
Greek	Indo-European	Athens	Greece	Europe	96[b]	4
Lebanese Christians	Afroasiatic	Beirut, Mount Lebanon, North Lebanon	Lebanon	Near East	52	2
Lebanese Druze	Afroasiatic	Mount Lebanon	Lebanon	Near East	31	
Lebanese Muslims	Afroasiatic	Beirut, Bekaa, North and South Lebanon	Lebanon	Near East	43	2
Yemen	Afroasiatic	Bayda, Dhamar, Marib, Sanaa	Yemen	Near East	20	

[a]Number of individuals
[b]2.5M SNPs extracted from sequence data

Table S2. African-Eurasian admixture time (generations ago) inferred using ALDER.

References	Laal	Sara	Toubou	Amhara
British/Fula	-	155 (±22), z=7.21	49 (±5), z=9.49	90 (±4), z=18.65
British/Ga-Adangbe	190 (±58), z=2.71	146 (±20), z=7.2	48 (±5), z=9.42	90 (±4), z=18.65
British/Igbo	193 (±55), z=2.81	143 (±20), z=7.29	48 (±5), z=9.07	90 (±4), z=18.52
British/Jola	174 (±53), z=2.74	154 (±19), z=8.06	49 (±5), z=9.58	90 (±4), z=17.91
British/Mandinka	196 (±57), z=2.72	151 (±20), z=7.68	49 (±5), z=9.19	91 (±4), z=18.42
British/Wolloff	205 (±48), z=3.13	154 (±19), z=7.96	49 (±5), z=9.27	90 (±4), z=17.97
British/Yoruba	222 (±68), z=2.41	149 (±20), z=7.29	47 (±5), z=9.03	90 (±4), z=18.76
Greek/Fula	-	158 (±20), z=7.91	48 (±5), z=9.27	90 (±4), z=19.53
Greek/Ga-Adangbe	193 (±51), z=3.15	148 (±19), z=7.65	47 (±5), z=9.19	89 (±4), z=19.51
Greek/Igbo	194 (±50), z=3.14	144 (±18), z=7.9	47 (±5), z=8.85	90 (±4), z=19.36
Greek/Jola	180 (±48), z=3.12	160 (±18), z=8.7	48 (±5), z=9.4	90 (±4), z=18.72
Greek/Mandinka	202 (±51), z=3.1	155 (±19), z=7.97	48 (±5), z=9.01	90 (±4), z=19.26
Greek/Wolloff	210 (±44), z=3.55	159 (±19), z=8.18	48 (±5), z=9.05	90 (±4), z=18.78
Greek/Yoruba	222 (±62), z=2.69	153 (±19), z=8.13	46 (±5), z=8.78	90 (±4), z=19.55
Gujarati/Fula	-	187 (±24), z=5.85	46 (±5), z=8.61	89 (±3), z=26.25
Gujarati/Ga-Adangbe	-	164 (±20), z=7.17	46 (±5), z=8.84	89 (±3), z=24.73
Gujarati/Igbo	-	157 (±19), z=6.96	45 (±5), z=8.35	89 (±3), z=24.32
Gujarati/Jola	-	183 (±23), z=6.01	47 (±5), z=9.05	89 (±3), z=23.78
Gujarati/Mandinka	-	175 (±21), z=6.02	47 (±5), z=8.55	90 (±3), z=24.93
Gujarati/Wolloff	-	184 (±22), z=6.36	46 (±5), z=8.82	89 (±3), z=24.1
Gujarati/Yoruba	-	164 (±20), z=6.83	45 (±5), z=8.21	89 (±3), z=24.44
Han/Fula	-	177 (±40), z=3.55	46 (±6), z=7.32	93 (±5), z=14.78
Han/Ga-Adangbe	-	160 (±28), z=5.28	45 (±6), z=7.78	92 (±5), z=15.05
Han/Igbo	-	149 (±26), z=5.73	45 (±6), z=7.26	91 (±5), z=15.06
Han/Jola	-	165 (±25), z=5.54	47 (±6), z=7.72	93 (±5), z=14.4
Han/Mandinka	-	174 (±28), z=4.83	46 (±6), z=7.38	93 (±5), z=15.12
Han/Wolloff	-	181 (±31), z=4.71	46 (±6), z=7.67	93 (±5), z=14.24
Han/Yoruba	-	162 (±33), z=4.73	44 (±6), z=7.25	92 (±5), z=14.99
Lebanese_Christian/Fula	-	158 (±22), z=7.2	48 (±5), z=8.71	91 (±4), z=18.67
Lebanese_Christian/Ga-Adangbe	199 (±54), z=2.98	148 (±19), z=7.77	47 (±5), z=8.64	90 (±4), z=18.69
Lebanese_Christian/Igbo	202 (±54), z=2.94	145 (±19), z=7.84	46 (±6), z=8.34	91 (±4), z=18.64
Lebanese_Christian/Jola	191 (±54), z=2.78	160 (±20), z=7.77	48 (±5), z=8.86	91 (±4), z=18.05
Lebanese_Christian/Mandinka	215 (±56), z=2.87	156 (±21), z=7.1	48 (±6), z=8.48	91 (±4), z=18.32
Lebanese_Christian/Wolloff	218 (±46), z=3.41	160 (±20), z=7.98	47 (±6), z=8.52	91 (±4), z=18
Lebanese_Christian/Yoruba	226 (±63), z=2.66	153 (±19), z=8.23	46 (±6), z=8.25	91 (±4), z=18.75
Lebanese_Druze/Fula	-	164 (±25), z=6.52	47 (±6), z=8.55	89 (±3), z=20.73
Lebanese_Druze/Ga-Adangbe	206 (±57), z=2.88	149 (±20), z=7.33	46 (±5), z=8.5	89 (±4), z=20.11
Lebanese_Druze/Igbo	210 (±56), z=2.9	146 (±18), z=7.92	46 (±6), z=8.22	89 (±4), z=20.05
Lebanese_Druze/Jola	195 (±52), z=2.92	164 (±21), z=7.61	48 (±5), z=8.7	90 (±4), z=19.42
Lebanese_Druze/Mandinka	219 (±56), z=2.88	160 (±23), z=7	47 (±6), z=8.31	90 (±4), z=19.76
Lebanese_Druze/Wolloff	221 (±46), z=3.43	164 (±22), z=7.45	47 (±6), z=8.37	90 (±4), z=19.29
Lebanese_Druze/Yoruba	232 (±68), z=2.5	154 (±19), z=8.09	46 (±6), z=8.12	90 (±3), z=20.29
Lebanese_Muslim/Fula	-	161 (±23), z=7.14	48 (±5), z=9.11	90 (±4), z=20.71
Lebanese_Muslim/Ga-Adangbe	208 (±54), z=3.05	150 (±20), z=7.43	47 (±5), z=9.02	89 (±4), z=20.24
Lebanese_Muslim/Igbo	211 (±54), z=3.06	147 (±19), z=7.7	47 (±5), z=8.68	89 (±4), z=20.02
Lebanese_Muslim/Jola	196 (±52), z=2.99	163 (±20), z=8.21	48 (±5), z=9.2	90 (±4), z=19.62
Lebanese_Muslim/Mandinka	223 (±54), z=3.06	158 (±21), z=7.36	48 (±5), z=8.79	90 (±4), z=19.91
Lebanese_Muslim/Wolloff	225 (±44), z=3.66	163 (±21), z=7.73	48 (±5), z=8.83	90 (±4), z=19.46
Lebanese_Muslim/Yoruba	238 (±64), z=2.74	154 (±20), z=7.67	46 (±5), z=8.53	90 (±4), z=20.27
Yemen/Fula	-	158 (±27), z=5.75	48 (±6), z=8	89 (±3), z=23.03
Yemen/Ga-Adangbe	189 (±45), z=3.38	145 (±21), z=6.85	46 (±6), z=7.94	89 (±3), z=22.21
Yemen/Igbo	195 (±47), z=3.26	145 (±20), z=7.25	46 (±6), z=7.74	89 (±3), z=22.21
Yemen/Jola	182 (±46), z=3.13	160 (±22), z=7.18	48 (±6), z=8.2	90 (±4), z=21.63
Yemen/Mandinka	202 (±48), z=3.22	155 (±24), z=6.39	48 (±6), z=7.88	90 (±4), z=21.9
Yemen/Wolloff	212 (±42), z=3.62	160 (±23), z=6.93	47 (±6), z=7.83	90 (±4), z=21.71
Yemen/Yoruba	212 (±55), z=2.93	150 (±21), z=7.11	46 (±6), z=7.59	89 (±3), z=22.58

Table S3. MALDER results (generations ago) allowing multiple admixture events.

	First event		Second event	
**Population**	**Reference**	**Time (sd), z-score**	**Reference**	**Time (sd), z-score**
Amhara	Laal/Greek	89(±4), z=26.28	-	-
Laal	Igbo/British	206(±42), z=4.86	-	-
Sara	Igbo/Greek	150(±15), z=9.81		
Toubou	Laal/British	109(±10), z=10.65	Laal/Greek	7(±2), z=5.12

Table S4. Y chromosomal haplogroup frequencies. Haplogroups were defined based on 636 Y-chromosomal SNPs overlapping with ISOGG markers (July 2016).

Haplogroup	Nigeria	Chad				Ethiopia					Kenya		Egypt	Lebanon			Yemen
	Yoruba	Laal	Sara	N'Djamena	Toubou	Amhara	Oromo	Somali	Wolayta	Gumuz	Luhya	Maasai	Egyptian	Christian	Druze	Muslim	Yemeni
A1b1b2b-M63	0	0	0.02	0.13	0	0.30	0.17	0	0.43	0.59	0	0.06	0.01	0	0	0	0
B-M181	0	0.54	0.10	0	0	0	0	0	0	0	0	0	0	0	0	0	0
B2-M247	0	0	0	0	0	0	0	0	0	0.41	0.02	0.06	0	0	0	0	0
B2a-M150	0	0	0.05	0.02	0	0	0	0	0	0	0.04	0	0	0	0	0	0
B2a1a1a1-M109	0	0	0.02	0.04	0	0	0	0	0	0	0	0	0	0	0	0	0
B2b-M112	0	0	0.02	0.02	0	0	0	0	0	0	0	0	0.01	0	0	0	0
E-M96	0.02	0	0	0	0	0	0	0	0.05	0	0.08	0.24	0	0	0	0	0
E1a-M132	0.07	0	0.02	0.05	0	0	0	0	0	0	0	0	0	0	0	0.02	0
E1b1a-V38	0	0	0	0	0	0.04	0	0	0.10	0	0	0	0	0	0	0	0
E1b1a1a1-P88	0	0	0	0	0	0	0	0	0	0	0	0	0.01	0	0	0	0
E1b1a1a1a-M58	0	0	0.02	0.02	0	0	0	0	0	0	0	0	0	0	0	0	0
E1b1a1a1b-M10	0	0.04	0.02	0	0.03	0	0	0	0	0	0	0	0	0	0	0	0
E1b1a1a1c-L458	0	0	0	0	0	0	0	0	0	0	0	0	0.01	0	0	0	0
E1b1a1a1c1a-P86	0.44	0.04	0.23	0.11	0.01	0	0	0	0	0	0.64	0.06	0	0	0	0	0
E1b1a1a1c1b-L516	0	0.15	0.18	0.07	0.01	0	0	0	0	0	0	0	0	0	0	0	0
E1b1a1a1d1-P277	0.47	0	0.07	0	0	0	0	0	0	0	0.14	0.12	0.02	0	0	0	0.05
E1b1b-PF1942	0	0	0	0.02	0.04	0.22	0.22	0	0.19	0	0.08	0.47	0	0	0	0.05	0.05
E1b1b1a1-PF2186	0	0	0.07	0.15	0.21	0.15	0.22	0.88	0.05	0	0	0	0.34	0.04	0.03	0	0
E1b1b1b1a1-M183	0	0	0	0.02	0.03	0	0	0	0	0	0	0	0.05	0	0.16	0.02	0
E1b1b1b2a-M123	0	0	0	0	0	0.04	0.06	0	0.19	0	0	0	0.09	0.02	0	0	0.20
E2-M75	0	0	0.02	0	0	0	0	0	0	0	0	0	0.01	0	0	0	0
G1-M285	0	0	0	0	0	0	0	0	0	0	0	0	0	0.06	0	0.02	0
G2-PF3138	0	0	0	0	0	0	0	0	0	0	0	0	0	0.08	0	0.02	0
G2a-PF3112	0	0	0	0	0	0	0	0	0	0	0	0	0	0	0.03	0.02	0.05
G2a2b-S126	0	0	0	0	0	0	0	0	0	0	0	0	0.05	0.06	0	0.12	0
G2b1-M377	0	0	0	0	0	0	0	0	0	0	0	0	0.01	0.02	0	0.02	0
H1-M69	0	0	0	0	0	0	0	0	0	0	0	0	0	0	0.06	0	0
I2-S329	0	0	0	0	0	0	0	0	0	0	0	0	0	0.02	0	0	0
J1-PF4782	0	0	0	0.13	0.01	0.11	0.33	0	0	0	0	0	0.20	0.08	0	0.10	0.60
J2-PF4908	0	0	0	0	0	0	0	0	0	0	0	0	0.01	0.06	0	0	0
J2a1-L26	0	0	0	0	0	0.07	0	0.06	0	0	0	0	0.05	0.17	0.29	0.29	0
J2b-PF4939	0	0	0	0	0	0.04	0	0	0	0	0	0	0	0	0	0	0
L1-M22	0	0	0	0	0	0	0	0	0	0	0	0	0	0.08	0.03	0	0
L1a1-M27	0	0	0	0	0	0	0	0	0	0	0	0	0.01	0	0.10	0.07	0
L1b1-M349	0	0	0	0	0	0	0	0	0	0	0	0	0	0.04	0	0	0
Q-M242	0	0	0	0	0	0	0	0	0	0	0	0	0	0.02	0	0.02	0
R1a1a1-M417	0	0	0	0	0	0	0	0	0	0	0	0	0.01	0	0	0	0
R1b1a-L761	0	0.23	0.20	0.15	0.34	0	0	0	0	0	0	0	0.03	0	0.03	0	0
R1b1a1a-P297	0	0	0	0	0	0	0	0	0	0	0	0	0.02	0	0	0	0
R1b1a1a2-L500	0	0	0	0	0	0	0	0	0	0	0	0	0.02	0.15	0.16	0	0
R1b1a1a2a1a-S128	0	0	0	0	0	0	0	0	0	0	0	0	0.01	0.04	0.03	0.05	0
R2a-P267	0	0	0	0	0	0	0	0	0	0	0	0	0	0	0	0.02	0
T-M272	0	0	0	0	0	0	0	0	0	0	0	0	0	0.02	0	0	0
T1a-PF5662	0	0	0	0.09	0.31	0.04	0	0.06	0	0	0	0	0.02	0.06	0.06	0.14	0.05
**No. of samples**	**43**	**26**	**61**	**55**	**71**	**27**	**18**	**16**	**21**	**17**	**50**	**17**	**99**	**52**	**31**	**42**	**20**

Table S5. Admixture f_3 statistics *(Test; Ref1, Yoruba)* where Ref1 is a present-day non sub-Saharan African population.

		Toubou			Amhara		
**Reference 1**	**Region**	**f3 values**	**S.E.**	**z-score**	**f3 values**	**S.E.**	**z-score**
Sardinian	Europe	-0.014559	0.000365	-39.84	-0.026489	0.000366	-72.309
Bergamo	Europe	-0.013812	0.000363	-38.085	-0.02557	0.00037	-69.149
Cypriot	Near East	-0.013104	0.000369	-35.502	-0.025539	0.000365	-70.001
Spanish_North	Europe	-0.014066	0.000396	-35.564	-0.025537	0.000425	-60.125
Basque	Europe	-0.013985	0.000369	-37.924	-0.025435	0.000373	-68.149
Lebanese_Christian	Near East	-0.012978	0.000341	-38.025	-0.025404	0.000315	-80.534
Tuscan	Europe	-0.013329	0.000384	-34.665	-0.025246	0.000401	-62.954
Greek	Europe	-0.01327	0.000352	-37.676	-0.025239	0.000333	-75.683
Georgian_Jew	Near East	-0.012718	0.000383	-33.196	-0.025085	0.000416	-60.311
Albanian	Europe	-0.013315	0.000397	-33.524	-0.025003	0.00041	-60.956
French_South	Europe	-0.013644	0.000384	-35.574	-0.024881	0.000404	-61.578
Iraqi_Jew	Near East	-0.01264	0.000384	-32.903	-0.02484	0.000403	-61.697
Yemenite_Jew	Near East	-0.012205	0.000367	-33.294	-0.024829	0.000389	-63.876
Armenian	Near East	-0.012636	0.000372	-34.011	-0.024781	0.000378	-65.59
Bulgarian	Europe	-0.013256	0.000377	-35.181	-0.024739	0.000383	-64.622
Italian_South	Europe	-0.013833	0.000537	-25.758	-0.024663	0.000632	-39.018
French	Europe	-0.013391	0.000363	-36.905	-0.024597	0.000362	-68
Croatian	Europe	-0.013172	0.00038	-34.623	-0.024539	0.000416	-59.045
English	Europe	-0.013171	0.000382	-34.466	-0.024325	0.000397	-61.341
Czech	Europe	-0.013014	0.000378	-34.396	-0.024285	0.000386	-62.953
Orcadian	Europe	-0.013003	0.000377	-34.53	-0.024245	0.000387	-62.609
British	Europe	-0.013094	0.000358	-36.543	-0.024234	0.00036	-67.368
Spanish	Europe	-0.013355	0.000351	-38.02	-0.024169	0.000342	-70.719
Georgian	Caucasus	-0.012282	0.000378	-32.491	-0.024142	0.000381	-63.354
Hungarian	Europe	-0.012911	0.000361	-35.785	-0.024109	0.000364	-66.185
Abkhasian	Caucasus	-0.012216	0.000378	-32.332	-0.024098	0.000399	-60.468
Saudi	Near East	-0.011961	0.000366	-32.66	-0.024089	0.000384	-62.666
Druze	Near East	-0.012417	0.000345	-36.019	-0.02407	0.000338	-71.197
Scottish	Europe	-0.013019	0.000413	-31.516	-0.024065	0.000451	-53.381
Icelandic	Europe	-0.013039	0.000384	-33.957	-0.024047	0.000396	-60.76
Norwegian	Europe	-0.013007	0.000381	-34.165	-0.024037	0.000398	-60.465
Lebanese_Druze	Near East	-0.012339	0.00035	-35.285	-0.024037	0.000336	-71.614
BedouinB	Near East	-0.012134	0.000339	-35.839	-0.024031	0.00034	-70.607
Sicilian	Europe	-0.012809	0.000371	-34.547	-0.023986	0.000373	-64.363
Lithuanian	Europe	-0.012663	0.000384	-33.003	-0.023674	0.000395	-59.922
Chechen	Caucasus	-0.012014	0.000371	-32.374	-0.023531	0.000388	-60.657
Ukrainian	Europe	-0.012641	0.000381	-33.179	-0.02347	0.0004	-58.668
Belarusian	Europe	-0.012575	0.000364	-34.508	-0.023319	0.000377	-61.854
Turkish	Near East	-0.012011	0.000339	-35.456	-0.023315	0.000324	-71.941
Adygei	Caucasus	-0.011843	0.000365	-32.418	-0.023298	0.000361	-64.504
Estonian	Europe	-0.012462	0.000374	-33.356	-0.022913	0.000393	-58.298
Lezgin	Caucasus	-0.011553	0.000369	-31.311	-0.022832	0.000374	-61.051
North_Ossetian	Caucasus	-0.011865	0.000368	-32.255	-0.022811	0.000377	-60.526
Lebanese_Muslim	Near East	-0.011667	0.000334	-34.957	-0.022727	0.000316	-71.857
Kumyk	Caucasus	-0.011635	0.000374	-31.073	-0.02263	0.00038	-59.601
Balkar	Caucasus	-0.011489	0.000369	-31.104	-0.022566	0.000386	-58.497
Russian	Europe	-0.012159	0.000361	-33.643	-0.022544	0.000369	-61.132
Finnish	Europe	-0.012297	0.000385	-31.955	-0.022498	0.000419	-53.694
Iranian	Near East	-0.011122	0.000362	-30.736	-0.022061	0.000381	-57.84
Lebanese	Near East	-0.011236	0.000349	-32.225	-0.021939	0.000351	-62.511
Yemeni	Near East	-0.010514	0.000321	-32.709	-0.02142	0.000299	-71.645
Syrian	Near East	-0.010962	0.000357	-30.686	-0.020863	0.000387	-53.905
Palestinian	Near East	-0.010886	0.00033	-33.034	-0.020802	0.00031	-67.094
Jordanian	Near East	-0.010636	0.000345	-30.841	-0.020252	0.000369	-54.911
Nogai	Caucasus	-0.010308	0.000371	-27.809	-0.019833	0.00039	-50.88
Tajik_Pomiri	central south asia	-0.009977	0.000367	-27.185	-0.019561	0.000385	-50.805
BedouinA	Near East	-0.010237	0.00032	-31.946	-0.018794	0.000312	-60.297
Balochi	central south asia	-0.009303	0.000352	-26.446	-0.018555	0.000361	-51.426
Brahui	central south asia	-0.009232	0.000348	-26.496	-0.018242	0.000355	-51.453
Kalash	central south asia	-0.009076	0.000389	-23.338	-0.017955	0.000422	-42.503
Pathan	central south asia	-0.009038	0.000348	-25.992	-0.017829	0.000361	-49.385
Cochin_Jew	Near East	-0.009278	0.00038	-24.416	-0.017737	0.000434	-40.889
Makrani	central south asia	-0.008611	0.000341	-25.229	-0.017194	0.000354	-48.515
Sindhi	central south asia	-0.008649	0.000342	-25.254	-0.017021	0.000372	-45.77
Burusho	central south asia	-0.008692	0.000343	-25.347	-0.016624	0.000358	-46.447
Uzbek	East Asia	-0.008837	0.000361	-24.499	-0.016574	0.000405	-40.876
GujaratiA	central south asia	-0.008589	0.000369	-23.264	-0.016445	0.000434	-37.884

Table S5 (continued). Admixture f_3 statistics *(Test; Ref1, Yoruba)* where Ref1 is a present-day non sub-Saharan African population.

		Toubou			Amhara		
**Reference 1**	**Region**	**f3 values**	**S.E.**	**z-score**	**f3 values**	**S.E.**	**z-score**
GujaratiB	central south asia	-0.008189	0.00039	-20.987	-0.015728	0.000443	-35.483
EgyptianA	North Africa	-0.009115	0.000306	-29.79	-0.015626	0.000312	-50.052
Mansi	East Asia	-0.008356	0.000399	-20.937	-0.015385	0.000469	-32.797
EgytpianB	North Africa	-0.008726	0.000283	-30.818	-0.015107	0.000246	-61.381
Gujarati	central south asia	-0.007651	0.000338	-22.632	-0.014868	0.000364	-40.859
Hazara	central south asia	-0.007893	0.000366	-21.581	-0.014858	0.000413	-36.017
Uygur	central south asia	-0.007818	0.000364	-21.456	-0.014844	0.000421	-35.284
GujaratiC	central south asia	-0.007571	0.000395	-19.145	-0.014788	0.000457	-32.388
Punjabi	central south asia	-0.007362	0.000359	-20.532	-0.014278	0.000417	-34.274
GujaratiD	central south asia	-0.00739	0.000385	-19.186	-0.014268	0.000452	-31.581
Tubalar	East Asia	-0.007636	0.000387	-19.752	-0.013625	0.000457	-29.83
Even	East Asia	-0.007523	0.000391	-19.259	-0.01355	0.00047	-28.834
Selkup	East Asia	-0.007504	0.000393	-19.075	-0.013386	0.000481	-27.814
Bengali	central south asia	-0.007008	0.000385	-18.193	-0.013124	0.000438	-29.956
Kyrgyz	central south asia	-0.007343	0.000384	-19.146	-0.013113	0.000473	-27.721
Altaian	East Asia	-0.006764	0.000395	-17.113	-0.012378	0.000497	-24.928
Quechua	Native American	-0.006357	0.000454	-13.994	-0.01162	0.000573	-20.286
Bolivian	Native American	-0.006456	0.000433	-14.926	-0.011307	0.000571	-19.802
Kalmyk	East Asia	-0.006183	0.000401	-15.414	-0.011269	0.000494	-22.832
Yukagir	East Asia	-0.006395	0.000392	-16.317	-0.011107	0.000493	-22.542
Surui	Native American	-0.006167	0.000504	-12.229	-0.011103	0.000677	-16.406
Tunisian	North Africa	-0.008142	0.000331	-24.62	-0.011078	0.000383	-28.931
Tuvinian	East Asia	-0.006255	0.000404	-15.487	-0.011065	0.000508	-21.785
Zapotec	Native American	-0.00641	0.000435	-14.743	-0.010943	0.000574	-19.073
Pima	Native American	-0.005948	0.000461	-12.895	-0.010651	0.000596	-17.865
Yakut	East Asia	-0.006216	0.000403	-15.434	-0.010639	0.000508	-20.952
Karitiana	Native American	-0.006142	0.000479	-12.813	-0.010628	0.000645	-16.486
Mayan	Native American	-0.006023	0.000421	-14.317	-0.010598	0.000559	-18.964
Mixe	Native American	-0.00613	0.000457	-13.404	-0.010559	0.000612	-17.253
Dolgan	East Asia	-0.006012	0.000445	-13.516	-0.010505	0.000575	-18.266
Mixtec	Native American	-0.005738	0.000439	-13.068	-0.009851	0.000588	-16.76
Thule	East Asia	-0.006153	0.001678	-3.667	-0.009729	0.002297	-4.236
Piapoco	Native American	-0.005517	0.000458	-12.037	-0.009676	0.000642	-15.078
Kusunda	East Asia	-0.005242	0.000409	-12.822	-0.0093	0.000512	-18.163
Itelmen	East Asia	-0.005389	0.000449	-11.999	-0.009144	0.000606	-15.093
Oroqen	East Asia	-0.005094	0.000422	-12.058	-0.009079	0.00055	-16.502
Mongola	East Asia	-0.005126	0.000432	-11.865	-0.009008	0.000563	-16.009
Mozabite	North Africa	-0.007802	0.000291	-26.781	-0.008966	0.000344	-26.057
Nganasan	East Asia	-0.005211	0.000445	-11.72	-0.008881	0.000592	-14.992
Koryak	East Asia	-0.005165	0.000435	-11.885	-0.008826	0.000573	-15.406
Xibo	East Asia	-0.005129	0.000429	-11.946	-0.008775	0.000549	-15.987
Algerian	North Africa	-0.007087	0.000352	-20.136	-0.008766	0.000457	-19.17
Nivkh	East Asia	-0.004748	0.00049	-9.687	-0.008761	0.000652	-13.43
Daur	East Asia	-0.005122	0.000415	-12.353	-0.008711	0.000533	-16.333
Tu	East Asia	-0.005086	0.000412	-12.348	-0.00865	0.000527	-16.421
Han_NChina	East Asia	-0.005099	0.00042	-12.15	-0.008563	0.000537	-15.944
Thai	East Asia	-0.005003	0.000412	-12.153	-0.00853	0.000519	-16.439
Hezhen	East Asia	-0.005014	0.000413	-12.135	-0.008519	0.000557	-15.302
Ulchi	East Asia	-0.004847	0.000415	-11.692	-0.008374	0.000546	-15.35
Korean	East Asia	-0.004918	0.000442	-11.136	-0.008306	0.000577	-14.404
Cambodian	East Asia	-0.004927	0.000411	-11.995	-0.00829	0.000527	-15.731
Tujia	East Asia	-0.004867	0.000426	-11.429	-0.008161	0.000538	-15.18
Miao	East Asia	-0.004718	0.000432	-10.913	-0.008154	0.000554	-14.711
Han	East Asia	-0.00476	0.000412	-11.563	-0.008064	0.000526	-15.327
Japanese	East Asia	-0.004718	0.000419	-11.26	-0.008058	0.000538	-14.972
Dai	East Asia	-0.004716	0.000426	-11.063	-0.007908	0.000541	-14.621
Yi	East Asia	-0.004648	0.00042	-11.063	-0.007863	0.000532	-14.772
Atayal	East Asia	-0.004587	0.000454	-10.101	-0.007837	0.000586	-13.365
She	East Asia	-0.004784	0.000432	-11.066	-0.007781	0.000566	-13.751
Ami	East Asia	-0.00458	0.000443	-10.331	-0.007733	0.000566	-13.663
Naxi	East Asia	-0.004585	0.000422	-10.856	-0.007716	0.000532	-14.493
Lahu	East Asia	-0.004561	0.000433	-10.533	-0.007672	0.000543	-14.131
Kinh	East Asia	-0.004431	0.000434	-10.201	-0.007414	0.000549	-13.5
Saharawi	North Africa	-0.007584	0.000339	-22.367	-0.00733	0.000448	-16.35
Botocudo	Native American	-0.003583	0.000605	-5.927	-0.005898	0.000835	-7.061
Bougainville	Oceanian	-0.00311	0.000444	-6.997	-0.005312	0.000658	-8.074
Australian	Oceanian	-0.002694	0.000506	-5.321	-0.004905	0.000753	-6.517
Papuan	Oceanian	-0.00236	0.000474	-4.978	-0.004038	0.000691	-5.84

Table S6. Admixture f_3 statistics *(Test; Ref1, Yoruba)* where Ref1 is an ancient population.

		Toubou			Amhara		
**Reference 1**	**Period and region**	**f3 values**	**S.E.**	**z-score**	**f3 values**	**S.E.**	**z-score**
LBK_EN	Early Neolithic European	-0.014918	0.000428	-34.818	-0.028068	0.000456	-61.539
Spain_EN	Early Neolithic European	-0.015126	0.00046	-32.861	-0.027009	0.000526	-51.35
HungaryGamba_EN	Early Neolithic European	-0.015156	0.000475	-31.934	-0.0283	0.000523	-54.072
Spain_MN	Middle Neolithic European	-0.014369	0.000486	-29.542	-0.025615	0.000574	-44.601
Unetice_EBA	Bronze Age Euopean	-0.012449	0.000441	-28.227	-0.023412	0.000497	-47.153
Bell_Beaker_LN	Late Neolithic European	-0.012543	0.000447	-28.031	-0.023304	0.000535	-43.551
Stuttgart	Early Neolithic European	-0.014762	0.000528	-27.958	-0.027612	0.000668	-41.362
Motala_HG	Hunter-gatherer European	-0.011612	0.000447	-25.953	-0.020525	0.000567	-36.22
Corded_Ware_LN	Late Neolithic European	-0.011707	0.000492	-23.795	-0.022564	0.00058	-38.892
Yamnaya	Bronze Age Eurasian	-0.010322	0.000439	-23.528	-0.020166	0.000499	-40.401
HungaryGamba_BA	Bronze Age Euopean	-0.012701	0.000575	-22.093	-0.023871	0.000696	-34.319
Iceman	Copper Age European	-0.01358	0.000621	-21.884	-0.025223	0.0008	-31.542
Loschbour	Hunter-gatherer European	-0.012448	0.000571	-21.783	-0.021552	0.000739	-29.175
SwedenSkoglund_NHG	Hunter-gatherer European	-0.012629	0.00059	-21.4	-0.02203	0.000786	-28.013
Esperstedt_MN	Middle Neolithic European	-0.014963	0.000713	-20.976	-0.028653	0.000892	-32.117
BenzigerodeHeimburg_LN	Late Neolithic European	-0.011048	0.000538	-20.541	-0.021253	0.000677	-31.405
Halberstadt_LBA	Bronze Age Euopean	-0.012164	0.000602	-20.191	-0.023085	0.000794	-29.065
Baalberge_MN	Middle Neolithic European	-0.014442	0.000733	-19.707	-0.02697	0.000848	-31.817
Alberstedt_LN	Late Neolithic European	-0.012738	0.00066	-19.304	-0.023392	0.000813	-28.776
SwedenSkoglund_MN	Middle Neolithic European	-0.013857	0.000723	-19.157	-0.025893	0.000931	-27.803
HungaryGamba_CA	Copper Age European	-0.014009	0.000757	-18.514	-0.026447	0.000944	-28.006
HungaryGamba_HG	Hunter-gatherer European	-0.012531	0.000684	-18.317	-0.02242	0.000904	-24.795
LaBrana1	Hunter-gatherer European	-0.011231	0.00062	-18.101	-0.01938	0.000827	-23.437
HungaryGamba_IA	Iron Age European	-0.012442	0.000695	-17.91	-0.023381	0.000936	-24.969
Samara_HG	Hunter-gatherer European	-0.011103	0.000722	-15.389	-0.018467	0.000969	-19.06
Starcevo_EN	Early Neolithic European	-0.015168	0.000993	-15.279	-0.028631	0.001227	-23.326
Karelia_HG	Hunter-gatherer European	-0.009448	0.000646	-14.621	-0.01781	0.000804	-22.161
Kostenki14	Paleolithic Eurasian	-0.008015	0.000688	-11.651	-0.014527	0.00089	-16.315
MA1	Paleolithic Eurasian	-0.007973	0.000692	-11.515	-0.015407	0.000933	-16.508
Karsdorf_LN	Late Neolithic European	-0.012201	0.001301	-9.375	-0.022034	0.001615	-13.643
Ust_Ishim	Paleolithic Eurasian	-0.002688	0.000567	-4.745	-0.003908	0.000806	-4.846
